# A new *p*-value based multiple testing procedure for generalized linear models

**DOI:** 10.1007/s11222-025-10600-2

**Published:** 2025-03-16

**Authors:** Joseph Rilling, Cheng Yong Tang

**Affiliations:** Department of Statistics, Operations, and Data Science, Temple University, Philadelphia, PA 19122 USA

**Keywords:** False discovery rate, Model-X knockoff, Paired estimators, *p*-values based multiple testing, Random row permutations

## Abstract

This study introduces a novel *p*-value-based multiple testing approach tailored for generalized linear models. Despite the crucial role of generalized linear models in statistics, existing methodologies face obstacles arising from the heterogeneous variance of response variables and complex dependencies among estimated parameters. Our aim is to address the challenge of controlling the false discovery rate (FDR) amidst arbitrarily dependent test statistics. Through the development of efficient computational algorithms, we present a versatile statistical framework for multiple testing. The proposed framework accommodates a range of tools developed for constructing a new model matrix in regression-type analysis, including random row permutations and Model-X knockoffs. We devise efficient computing techniques to solve the encountered non-trivial quadratic matrix equations, enabling the construction of paired *p*-values suitable for the two-step multiple testing procedure proposed by Sarkar and Tang (Biometrika 109(4): 1149–1155, 2022). Theoretical analysis affirms the properties of our approach, demonstrating its capability to control the FDR at a given level. Empirical evaluations further substantiate its promising performance across diverse simulation settings.

**Supplementary Information:**

The online version contains supplementary material available at 10.1007/s11222-025-10600-2.

## Introduction

In statistical testing, adjusting for the multiplicity of hypotheses of interest is of fundamental importance; see the overview in Benjamini (2010). The false discovery rate (FDR) is a crucial concept in this context, as introduced by the seminal work of Benjamini and Hochberg (1995). Compared with methods that control the family-wise error rate, those capable of controlling the FDR present a sound and less conservative approach for testing many hypotheses. These FDR controlling procedures are generally more powerful, making them compelling for making new discoveries. The Benjamini–Hochberg method (Benjamini and Hochberg 1995) is the most popular approach attempting to control the FDR. While results have been established showing the method controls the FDR under the settings of independence among the multiple test statistics and some specified positive dependence structures (Benjamini and Yekutieli 2001; Finner et al. 2007; Sarkar 2002), its properties under the general dependence of the testing statistics remain not fully understood. Therefore, a longstanding challenge in multiple testing methodology is how to handle arbitrary dependence between the test statistics.

There have been recent influential developments in methods for controlling the FDR using the knockoff methodology; see, among others, Barber and Candès (2015) and Candes et al. (2016). This framework involves constructing a new copy of the model matrix, allowing for the estimation of the false discovery proportion in various testing procedures. By controlling the estimated false discovery proportion, these methods have shown effectiveness in FDR control without specific assumptions about the dependence between test statistics. In contrast, a conventional technique by randomly permuting the rows of the model matrix provides a straightforward strategy, disrupting the association between covariates and response variables while preserving the between-component covariance of the covariates. However, while the random row permutation strategy can establish a null model with no covariate influencing the response variable, it has not been utilized in the literature to develop methodologies showcasing FDR control. Both random row permutation and knockoff approaches can be seen as attempts to regenerate a new copy of the model matrix. As a related method, rather than regenerating a new model matrix, Dai et al. (2023) recently introduced an approach based on data-splitting, obtaining “mirror statistics” to estimate the false discovery proportion. Subsequent methods developed in Dai et al. (2023) aim to control the estimated false discovery proportion and have been shown to effectively control the FDR.

Recently, building upon the knockoff copy of the model matrix, Sarkar and Tang (2022) proposed a new *p*-value-based approach as an alternative to estimating the false discovery proportion in the context of linear models. Their method leverages the properties of a set of paired *p*-values, enabling a new two-step procedure that employs Bonferroni’s method in the first step for screening and subsequently applies the Benjamini–Hochberg method in the second step. In the context of linear models, their approach is demonstrated to control the FDR under arbitrary dependence between covariates. In settings with lower FDR levels and fewer numbers of covariates, the method of Sarkar and Tang (2022) exhibits competitiveness in its power compared to knockoff methods based on estimated false discovery proportions.

In the realm of statistical modeling, the class of generalized linear models serves as a fundamental tool for understanding the relationship between covariates and responses in various domains. Multiple testing problems for generalized linear models are thus of key importance. However, relative to the extensive exploration in the context of linear models, this area remains less explored. The primary challenge, as discussed earlier, arises from the inherent difficulty of managing the dependence between test statistics, posing a significant obstacle in this area of research.

Our aim in this study is to develop a valid *p*-value-based multiple testing approach for generalized linear models that controls the FDR with arbitrarily dependent test statistics. Compared with linear models, there are substantial new challenges. Our analysis reveals that, for generalized linear models, the heterogeneous variance of the response variables leads to dependence between the estimated parameters, making the construction of paired *p*-values, as proposed by Sarkar and Tang (2022), inapplicable. To address the challenges at hand, we introduce a novel framework: a *p*-value-based multiple testing framework designed for generalized linear models with a generic regenerated model matrix. Our study makes crucial contributions. Firstly, we develop a specialized set of computing algorithms adept at efficiently solving a non-trivial quadratic matrix equation and facilitating the generation of paired *p*-values that comprehensively account for both the original model matrix and a generic regenerated one. These paired *p*-values are tailored to meet the necessary criteria for implementing the two-step multiple testing procedure proposed by Sarkar and Tang (2022). Remarkably, our framework is versatile, accommodating various practical techniques such as Model-X knockoffs and random row permutations to construct a new model matrix. As a promising outcome, our approach is capable of controlling the FDR at a given level for generalized linear models with arbitrary dependent model matrices. We substantiate our approach through theoretical validation and empirical evaluations, underscoring its competitiveness.

The rest of this article is organized as follows. Sect. 2 gives the needed background on generalized linear models and the proposed model setting. Section 3 provides a detailed exposition of our proposed methodology and main results. Simulation examples are reported in Sect. 4, followed by a real data example in Sect. 5. Technical proofs and additional numerical studies are presented in the Appendix.

## Overview

### Generalized linear models

Practically, broad types of response variables are common. For example, the outcome of interest may be binary (e.g. positive/negative diagnosis, win/lose a single game, credit card fraud detection) or count data (e.g. number of sales, number of car accidents, number of friends). In these situations, the generalized linear model (McCullagh and Nelder 1998) serves as the default tool for statistical inference.

Let $Y_1, \ldots , Y_n$ be independent random variables associated with covariates $\varvec{X}_{\textbf{1}}, \ldots , \varvec{X}_{\varvec{n}}$, where $\varvec{X}_{\varvec{i}} = (X_{i1}, \ldots , X_{id})^{ { {\textrm{T}}} }\in {\mathbb {R}}^d$ $(i = 1, \ldots , n)$. Generalized linear models assume that $Y_i$ belongs to the exponential family, and declare that the covariates $\varvec{X}_{\varvec{i}}$ are related to the response $Y_i$ through:

1 $$\begin{aligned} g(\mu _i)=\eta _i=\varvec{X}_i^{ { {\textrm{T}}} }\varvec{\beta }. \end{aligned}$$

Here, $\mu _i = {\mathbb {E}}(Y_i)$, $\varvec{\beta } \in {\mathbb {R}}^d$ represents the unknown model parameter, with its true value denoted as $\varvec{\beta }_{\textbf{0}}$, and $g(\cdot )$ is known as the link function. For example, in Gaussian linear models with continuous response and an identity link, we have $g(\mu ) = \mu$. In logistic regression with binary response, the logit link is often used, defined as $g(\mu ) = \text {logit}(\mu ) = \log \left( \mu /(1-\mu )\right)$. In Poisson regression with count-valued responses, the log-link is commonly employed, given by $g(\mu ) = \log (\mu )$.

In the regular case of generalized linear models, the maximum likelihood estimator $\hat{\varvec{\beta }}$ of $\varvec{\beta }$ is asymptotically normal: $\Gamma ^{-1/2}(\hat{\varvec{\beta }}-\varvec{\beta }_{\textbf{0}}) \sim N({\textbf{0}}_{\varvec{d}}, I_d)$ approximately as $n\rightarrow \infty$, where $I_d$ is the identity matrix of size *d*. Here, the covariance matrix is $\Gamma =(X^{ { {\textrm{T}}} }W X)^{-1}$, where $X=(\varvec{X}_{\textbf{1}},\dots , \varvec{X}_{\varvec{n}})^{ { {\textrm{T}}} }\in {{\mathbb {R}}}^{n\times d}$ is the model matrix, and $W=\textrm{diag}(w_1,\dots , w_n)$ with $w_i = (d\mu _i/d\eta _i)^2/ \text {var}(Y_i)$. The variance of the outcome $\text {var}(Y_i)$ relates to its mean $\mu _i$ through a connection called the mean variance-relationship. For instance, in logistic regression with binary $Y_i$ and probability of success/mean $\mu _i$, $\text {var}(Y_i) = \mu _i(1-\mu _i)$. In Poisson regression with count-valued $Y_i$, $\text {var}(Y_i) = \mu _i$. Hence, $\Gamma$ contains unknown parameters connected to $\mu _i$ (and, consequently, to the variance of $Y_i$), making variance estimation for $\hat{\varvec{\beta }}$ a necessity in practice. This relationship between mean, variance, and fitted values results in an estimator ${\hat{\Gamma }} = (X^T {\hat{W}} X)^{-1}$.

The special case of Gaussian linear models with a homogeneous variance assumption ($\text {var}(Y_i) = \sigma ^2$) has several advantages that make this subclass of generalized linear models easy to work with. Homogeneous variance linear models use the identity link function, which, combined with the assumed constant variance for $Y_i$, implies *W* is solely a function of $\sigma ^2$, is independent of $\varvec{X}_{\varvec{i}}$, and takes a constant value along the diagonal. As a result of these properties, homogeneous Gaussian linear models possess a statistical inference framework using known normal-related distributions such as the *t*, $\chi ^2$, and *F* distributions. In the broader class of generalized linear models, these properties and resulting advantages are not present. Our investigation reveals that the heterogeneous variances of $Y_i$ in *W* introduce intriguing new challenges and opportunities for multiple testing problems within the context of generalized linear models.

### Multiple testing with augmented model matrix

We are interested in testing $H_{0j}: \beta _j=0$ $(j=1,\dots , d)$. The setting we consider in this study is $n>2d$, inspired by that of Barber and Candès (2015) and Sarkar and Tang (2022).

Our setup begins with the introduction of an augmented model matrix. Let ${\tilde{X}}=(\tilde{\varvec{X}}_{\varvec{1}}, \dots , \tilde{\varvec{X}}_{\varvec{n}})^{ { {\textrm{T}}} }\in {{\mathbb {R}}}^{n\times d}$ be a regenerated model matrix that satisfies the following requirement for ${\tilde{X}}$.

#### Condition1 1

Conditioning on *X*, ${\tilde{X}}$ and *Y* are independent.

Examples of ${\tilde{X}}$ include knockoffs (Barber and Candès 2015; Candes et al. 2016) and random row permutations of the original *X*. Subsequently, we aim to fit the response variables $(Y_1, \ldots , Y_n)$ with the augmented matrix $(X, {\tilde{X}})$ through a working model:

2 $$\begin{aligned} g(\mu _i)=\eta _i&=\varvec{X}_{\varvec{i}}^{ { {\textrm{T}}} }\varvec{\theta }_{\textbf{1}}+\tilde{\varvec{X}}_{\varvec{i}}^{ { {\textrm{T}}} }\varvec{\theta }_{\textbf{2}} \end{aligned}$$

If model (1) holds true, then the true values of $\varvec{\theta } = (\varvec{\theta }_{\textbf{1}}^{ { {\textrm{T}}} }, \varvec{\theta }_{\textbf{2}}^{ { {\textrm{T}}} })^{ { {\textrm{T}}} }$ are $\varvec{\theta }_{\textbf{0}} = (\varvec{\theta }_{\textbf{10}}^{ { {\textrm{T}}} }, \varvec{\theta }_{\textbf{20}}^{ { {\textrm{T}}} })^{ { {\textrm{T}}} }$ with $\varvec{\theta }_{\textbf{10}} = \varvec{\beta }_{\textbf{0}}$ and $\varvec{\theta }_{\textbf{20}} = {\textbf{0}}_{\varvec{d}}$. As $n \rightarrow \infty$, the maximum likelihood estimator $\hat{\varvec{\theta }}$ is asymptotically normal: as $n\rightarrow \infty$

$$\begin{aligned} \Omega ^{-1/2}(\hat{\varvec{\theta }}-\varvec{\theta }_{\textbf{0}}) \sim N({\textbf{0}}_{\varvec{d}}, I_{2d}) \end{aligned}$$

where $\Omega =\{(X, {\tilde{X}})^{ { {\textrm{T}}} }W (X, {\tilde{X}})\}^{-1}$. As mentioned above, *W* contains unknown parameters related to the true values of $\mu _i$ and must be estimated as ${\hat{W}}$. Therefore, we estimate the variance for $\hat{\varvec{\theta }}$ as $\hat{\Omega }=\{(X, {\tilde{X}})^{ { {\textrm{T}}} }{{\hat{W}}} (X, {\tilde{X}})\}^{-1}$.

Let $$Z =\begin{bmatrix} I_d & P \\ I_d & V \end{bmatrix}$$ where $I_d$ is the identity matrix of size *d*, *P* and *V* are two $d\times d$ matrices. Since $\varvec{\theta }_{\textbf{0}}=(\varvec{\beta }_{\textbf{0}}^{ { {\textrm{T}}} }, {\textbf{0}}_{\varvec{d}})^{ { {\textrm{T}}} }$, $Z\varvec{\theta }_{\textbf{0}}=(\varvec{\beta }_{\textbf{0}}^{ { {\textrm{T}}} }, \varvec{\beta }_{\textbf{0}}^{ { {\textrm{T}}} })^{ { {\textrm{T}}} }$, and applying the transformation *Z* to the maximum likelihood estimates $Z\hat{\varvec{\theta }}=(\hat{\varvec{\beta }}_{\varvec{1}}^{ { {\textrm{T}}} }, \hat{\varvec{\beta }}_{\varvec{2}}^{ { {\textrm{T}}} })^{ { {\textrm{T}}} }$ creates a pair of two asymptotically unbiased estimators, $\hat{\varvec{\beta }}_{\varvec{1}}$ and $\hat{\varvec{\beta }}_{\varvec{2}}$, for $\varvec{\beta }_{\textbf{0}}$.

In the case of homoskedastic linear models, setting ${\tilde{X}}$ as the Fixed-X knockoff of Barber and Candès (2015), and using a special case of *Z* with $P=I_d$ and $V=-I_d$ gives the case considered in Sarkar and Tang (2022). This construction notably gives two independence properties: $\hat{\varvec{\beta }}_{\varvec{1}}$ and $\hat{\varvec{\beta }}_{\varvec{2}}$ are independent, and the covariance matrix of $\hat{\varvec{\beta }}_{\varvec{2}}$ is diagonal, i.e., the components of $\hat{\varvec{\beta }}_{\varvec{2}}$ are independent. Based on the *p*-values created from the *t* statistics constructed from the pair $(\hat{\varvec{\beta }}_{\varvec{1}}^{ { {\textrm{T}}} }, \hat{\varvec{\beta }}_{\varvec{2}}^{ { {\textrm{T}}} })^{ { {\textrm{T}}} }$, Sarkar and Tang (2022) proposed a *p*-valued based multiple testing procedure that controls the false discovery rate with arbitrary model matrix *X*.

For broad generalized linear models, because *W* includes the mean functions through the mean-variance relationship, the choice $P=I_d$ and $V=-I_d$ no longer ensures that $\hat{\varvec{\beta }}_{\varvec{1}}$ and $\hat{\varvec{\beta }}_{\varvec{2}}$ are independent for ${\tilde{X}}$ chosen as knockoff copies of *X* (see the illustration in Sect. S.6 in the supplementary material). Thus, results of Sarkar and Tang (2022) do not hold for broad general linear models.

## Methodology

To address these challenges, we propose a new procedure for multiple testing with generalized linear models. First, we construct a transformation matrix $Z$ that ensures desirable properties for the resulting paired estimators of $\varvec{\beta }$, given by $Z\hat{\varvec{\theta }} = (\hat{\varvec{\beta }}_{\varvec{1}}^{ { {\textrm{T}}} }, \hat{\varvec{\beta }}_{\varvec{2}}^{ { {\textrm{T}}} })^{ { {\textrm{T}}} }$. Next, we apply the multiple testing procedure of Sarkar and Tang (2022) to the $p$-values derived from the limiting distribution of the constructed paired estimators.

### Constructing a new transformation of ${{\hat{\theta }}}$

We write $${{\hat{\Omega }}}=\{(X, \tilde{X})^{ { {\textrm{T}}} }{\hat{W}}(X, \tilde{X})\}^{-1} = \begin{bmatrix} J & K \\ K^{ { {\textrm{T}}} }& N \end{bmatrix}$$, where all the blocks are of size $d\times d$, and *J* and *N* are symmetric. We can compute the variance as follows:

$$\begin{aligned}&\text {var}(\hat{\varvec{\beta }}_{\varvec{1}}^{ { {\textrm{T}}} }, \hat{\varvec{\beta }}_{\varvec{2}}^{ { {\textrm{T}}} })=\text {var}(Z\varvec{{\hat{\theta }}}) = Z\hat{\Omega }Z^{ { {\textrm{T}}} }\end{aligned}$$

We have two main objectives. The first is independence between $\hat{\varvec{\beta }}_{\varvec{1}}$ and $\hat{\varvec{\beta }}_{\varvec{2}}$. This goal translates to the restriction that $Z{{\hat{\Omega }}} Z^T$ must be a block diagonal matrix, ultimately requiring:

$$\begin{aligned}V = -(J+KP^{ { {\textrm{T}}} })(K^{ { {\textrm{T}}} }+ NP^{ { {\textrm{T}}} })^{-1}, \end{aligned}$$

or equivalently:

3 $$\begin{aligned} P = -(J+KV^{ { {\textrm{T}}} })(K^{ { {\textrm{T}}} }+ NV^{ { {\textrm{T}}} })^{-1}. \end{aligned}$$

In addition to independence between $\hat{\varvec{\beta }}_{\varvec{1}}$ and $\hat{\varvec{\beta }}_{\varvec{2}}$, the second objective for *Z* is to ensure that the components in the limiting distribution of $\hat{\varvec{\beta }}_{\varvec{2}}$ are mutually independent. To achieve this goal, the covariance matrix of $\hat{\varvec{\beta }}_{\varvec{2}}$ must be diagonal. That is, for some diagonal matrix $D\in {{\mathbb {R}}}^{d\times d}$, it must hold that:

4 $$\begin{aligned} J + VK^{ { {\textrm{T}}} }+ KV^{ { {\textrm{T}}} }+ VNV^{ { {\textrm{T}}} }= D \end{aligned}$$

The decision maker can specify the diagonal matrix $D$ as desired, as long as $D - J$ is symmetric and positive semi-definite, which is a requirement of the solver we use for (4). Here, we provide guidance, simulation evidence, and a suggestion. Since $H = D - J$ is symmetric, it is positive semi-definite if and only if all its eigenvalues are non-negative. If we set $D$ as a constant diagonal matrix, $D = \textrm{diag}(\delta )$, then the eigenvalues of $H$ are $\delta - \lambda _{J,i}, \forall i=1,\ldots ,d$, where $\lambda _{J,i}$ is the $i$-th eigenvalue of $J$. We can define the constant entry $\delta$ as a multiple of $J$’s maximum eigenvalue, $\delta = \delta _c \cdot \lambda _{J,\textrm{max}}$. By imposing the constraint $\delta _c \ge 1$, we ensure that $H$ has non-negative eigenvalues, making it positive semi-definite, ensuring that (4) satisfies the conditions required by the solving method we employ.

Simulation evidence on the choice of $\delta _c$ is provided in Sect. S.5. These simulations clearly show that a lower $\delta _c$ generally performs better. Since the diagonal matrix $D$ controls the variance of the second set of coefficients, it is intuitive that lower variances lead to increased power. The multiplicative constant $\delta _c = 1.1$ performs well across all settings considered, and thus we recommend this choice, which is also the setting used in the results presented in this paper. Exploring the optimal choice of $D$, which need not have constant entries along the diagonal, is an interesting and promising direction for future research.

Our task now revolves around finding *P* and *V* that satisfy (3) and (4), as detailed in Sect. 3.2. The main challenge turns out to be equivalent to solving a nontrivial quadratic matrix equation for *V*, subject to the constraint that *V* is symmetric. Plugging the found *V* into (3) then yields *P* and solves for *Z*.

### Implementing the transformation

We now elaborate on the algorithm used to estimate *Z*, which solves (3) and (4). Full details are provided in the supplementary material. We begin by solving (4) for *V*. Then *P* can be obtained through (3), completing the estimation of the desired transformation. The first equation of interest, (4), can be mapped to a continuous algebraic Riccati equation (CARE). This type of equation is common in infinite-horizon optimal control problems, where practitioners must set covariate values in the present to control the long-term behavior of the outcome of interest. In particular, the general form of a CARE is

5 $$\begin{aligned} F^{T}V+VF-VGV+H=0. \end{aligned}$$

where *G* and *H* are symmetric and positive semidefinite. Letting $H = D - J$, $F = -K^T$, $G= N$, (4) is then rewritten in a form of Continuous Algebraic Riccati Equation (CARE). There are several schemes available for solving CARE. In this study, we exploit the “Schur Method” of Laub (1979) to find the stabilizing solutions of (5), as detailed below. The Schur method constructs a Hamiltonian matrix $$\Phi = \begin{bmatrix} F & -G \\ -H & -F^T \end{bmatrix} \in {\mathbb {R}}^{2d\times 2d}$$. Under broad assumptions, there exists an orthogonal transformation $$U = \begin{bmatrix} U_{11} & U_{12} \\ U_{21} & U_{22} \end{bmatrix}$$ such that $U^T\Phi U = S$ where *S* is a quasi upper triangular matrix whose diagonal entries are the real parts of the eigenvalues $\lambda _{1}, \lambda _2,...,\lambda _{2d}$ of $\Phi$. For this constructed Hamiltonian, the stabilizing solution that we are looking for, *V*, is a function of two blocks of this orthogonal matrix *U*: $V = U_{21}U_{11}^{-1}$ (Arnold and Laub 1984). We carry out this procedure, constructing the Hamiltonian, finding the orthogonal matrix *U*, and estimating *V* using the “control” R package (Ubah 2017).

Finally, plug the found *V* into (3) to obtain *P*. The resulting $Z{\hat{\varvec{\theta }}}$ satisfies our two objectives as described in Sect. 3.1, and serves as the foundation for us to propose a new two-step *p*-value based procedure described in the following section.

### A paired *p*-value based multiple testing method

The proposed multiple testing procedure is built upon two key components: the paired estimator $Z\hat{\varvec{\theta }}=(\hat{\varvec{\beta }}_{\varvec{1}}^{ { {\textrm{T}}} }, \hat{\varvec{\beta }}_{\varvec{2}}^{ { {\textrm{T}}} })^{ { {\textrm{T}}} }$ established in Sects. 3.1 and 3.2, and the Bonferroni–Benjamini–Hochberg procedure outlined in Sarkar and Tang (2022) that incorporates paired *p*-values. As a whole, we name our method the “generalized two step” (GTS). We summarize the framework of our approach in Algorithms 1-3.

Algorithm 1 details the construction of the paired *p*-values: $\varvec{P^{(1)}} = (P^{(1)}_1,\ldots , P^{(1)}_d)$, and $\varvec{P^{(2)}}= (P^{(2)}_1,\ldots , P^{(2)}_d)$. Algorithm 2 uses these *p*-values to make decisions on variable significance; Theorem 1 shows that Algorithms 1 and 2 together asymptotically control the false discovery rate at level $\pi _0\alpha$, where $\pi _0$ is the proportion of null covariates among the original design *X*. Algorithm 3 is an adaptive procedure incorporating the estimated ${\hat{\pi }}_0$ to asymptotically control the false discovery rate at level $\alpha$ as shown in Theorem 2, leading to a more powerful procedure if used in placed of Algorithm 2. To facilitate a regular asymptotic analysis of the maximum likelihood estimator, our theory lets $n \rightarrow \infty$ while keeping *d* fixed. Proofs of the theorems are presented in the supplementary material.

**Algorithm 1:**
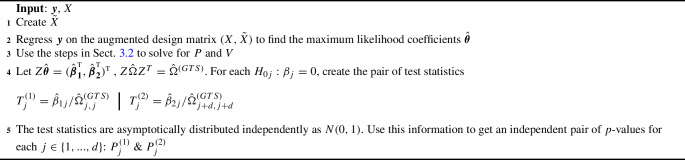
Creating Test Statistics and Valid *p*-values

**Algorithm 2:**
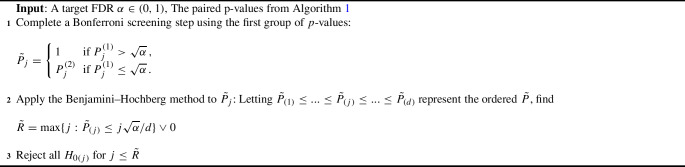
A Two-step Multiple Testing Procedure

**Algorithm 3:**
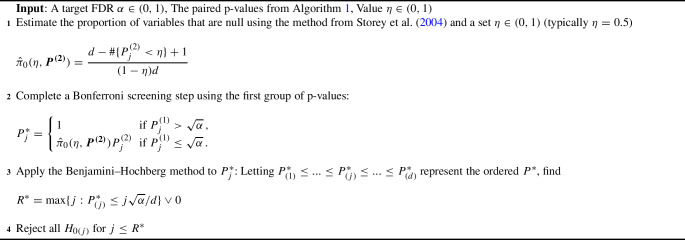
An Adaptive Two-step Multiple Testing Procedure

#### Theorem 1

The procedure using Algorithm 1 and Algorithm 2 asymptotically controls the FDR, denoted by FDR(*n*), at $\pi _0\alpha$ as $n\rightarrow \infty$, where $\pi _0$ is the proportion of the true nulls. Specifically,

$$\begin{aligned} \lim _{n \rightarrow \infty } \text {FDR}(n) \le \pi _0\alpha . \end{aligned}$$

#### Theorem 2

The adaptive procedure using Algorithms 1 and 3 asymptotically controls the FDR, denoted by FDR(*n*), at $\alpha$ as $n\rightarrow \infty$. Specifically,

$$\begin{aligned} \lim _{n \rightarrow \infty } \text {FDR}(n) \le \alpha . \end{aligned}$$

### Discussion

The strength of our method lies in its capacity to accommodate a wide spectrum of choices for ${\tilde{X}}$, ranging from intricate Model-X knockoffs to straightforward random row permutations. This adaptability allows researchers to make selections tailored to their specific data, research objectives, or experimental contexts, thereby enhancing versatility. An advantage of our approach is that it does not require custom procedures tailored to the specific creation of ${\tilde{X}}$ and the specific types of response variables. This generality establishes a unified *p*-value-based multiple testing procedure for generalized linear model as a valuable tool for researchers seeking insightful analyses of complex and diverse datasets.

Although the proposed method bears some resemblance to “screen-and-test” procedures used in higher-dimensional settings, our approach in this work is inherently low-dimensional. While extending our method to higher dimensions, such as scenarios where $d< n < 2d$ and even higher, is of great interest, it presents significant challenges. In particular, Theorems 1 and 2, which guarantee asymptotic control of the false discovery rate, fundamentally rely on the asymptotic distribution of generalized linear model coefficients. However, this approximation is unreliable when *n* is not substantially larger than *d*. As Portnoy (1988) argues, the asymptotic approximation is appropriate only when $n \ge (2d)^{3/2}$. In the case of logistic regression, for instance, it is generally practical that at least 10 events per variable are needed, yet Sur and Candès (2019) points out that this minimum threshold is often insufficient.

Moreover, the Schur method, which is central to our current framework, is inherently low-dimensional and tends to struggle, or even fail entirely, in higher-dimensional contexts. In theory, approaches that augment the response vector and design matrix, such as those proposed by Barber and Candès (2015) and Sarkar and Tang (2022), might extend the method to settings where $n < 2d$. However, such extensions are non-trivial and generally inadvisable due to both the limitations of asymptotic approximations and the instability of the Schur method in these dimensions.

## Simulations

### Overview

In all simulations, the covariates *X* are drawn from a multivariate normal distribution $N({\textbf{0}}_{\varvec{d}},\Sigma )$ where $\Sigma$ is a autoregressive matrix with coefficient of correlation $\rho$ and entries $\Sigma _{ij} = \rho ^{\mid i - j \mid }$. For these simulations, we set $\rho$ to a default value of 0.5 unless otherwise specified. For each simulation, we generate *n* observations, *d* covariates, and *k* ($k<d$) true nonzero coefficients. A nonzero coefficient $\beta _j$ has randomly assigned direction and magnitude $\beta _j = |\textrm{amplitude}|/\sqrt{n}$. In the following plots, amplitude varies along the *x*-axis. The desired FDR control is denoted as $\alpha$. At each amplitude, the simulations are repeated 250 times.

**Fig. 1:**
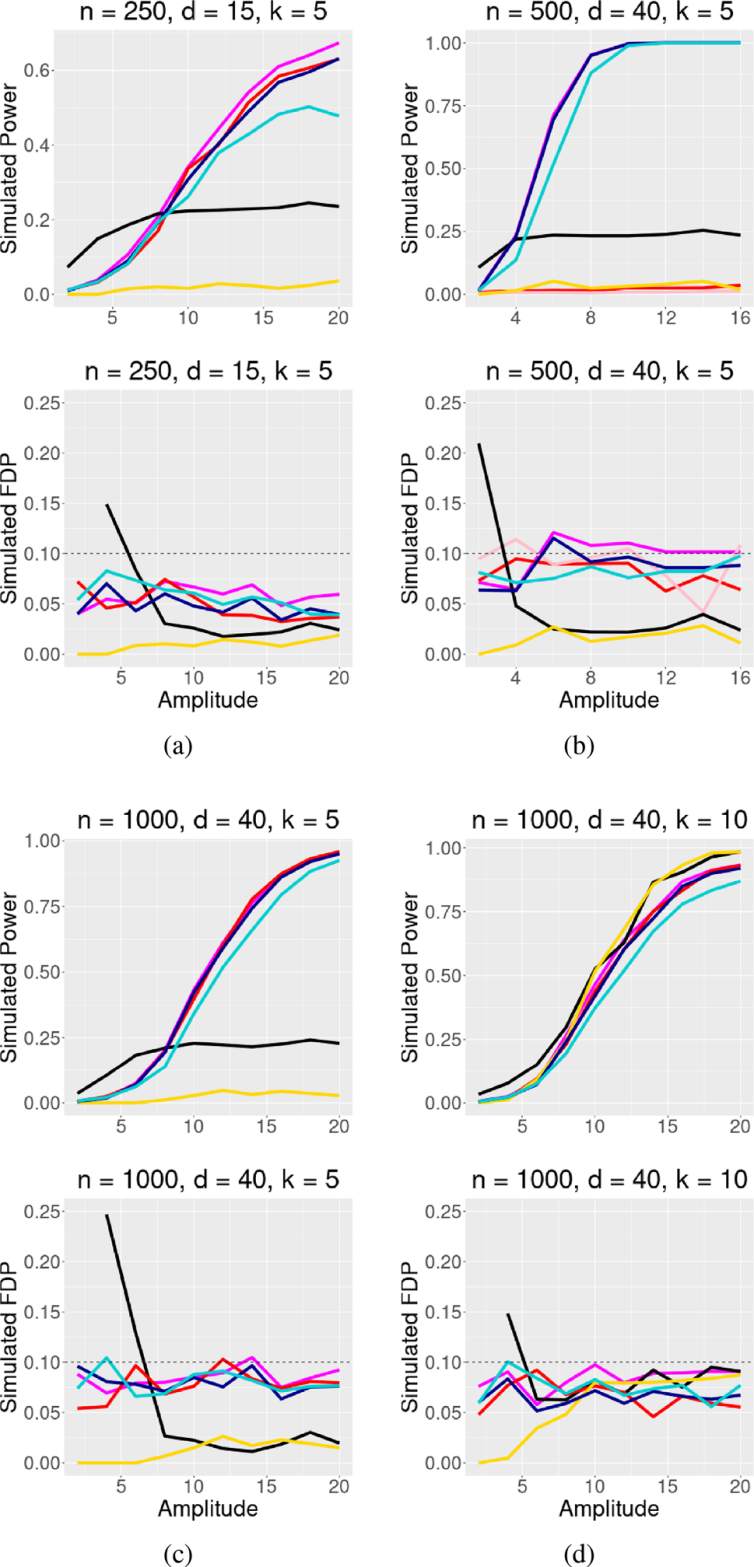
Logistic regression simulation with $\rho = 0.5$ and target FDR $\alpha = 0.1$. Signal amplitude varies on the x axis, and simulated power/FDR is along the y axis. The simulation compares the GTS method, Beta-2 Only method,AGTS method, GTS-Full Permutation, **Data Splitting** (black) and the Model-X knockoff filter

We present the performance of the proposed method in logistic regression and Poisson regression. Additional results pertaining to linear regression are available in the supplementary materials (Sect. S.7). In these analyses, we compare our method, along with its adaptive counterpart that adjusts with the estimated null proportion, against several competing approaches. Our comparisons include Model-X knockoffs and a method from Dai et al. (2023) based on data splitting.

Our approach and the data splitting method (referred to as DS in the results) introduced by Dai et al. (2023) share a common principle in that both methods generate two sets of independent estimates for each coefficient within a generalized linear model. A key distinction between our method and the approach of Dai et al. (2023) lies in the strategy for controlling false discoveries. Our method is *p*-value-based, while the approach of Dai et al. (2023) relies on estimating the false discovery proportion. Another distinction is how the two methods create independent estimates. The data splitting approach splits the set of observations into two non-overlapping groups, each containing half of the *n* observations, while our method introduces conditionally null covariates and adjusts for dependence via a linear transformation.

From the simulation study, we observe that our method outperforms Model-X knockoffs and the data splitting approach in settings with sparse signals and low FDR levels. However, in dense signal settings and/or situations with a higher FDR level, both Model-X knockoffs and the data splitting procedure tend to perform better. The crux of this phenomenon lies in the contrasting approaches employed by the aforementioned methods for selecting relevant variables. Our proposed method, unlike the knockoff filter and data splitting approaches, anchors its decisions on *p*-values that incorporate a comprehensive quantification of the associated uncertainties. The merits of using *p*-values become evident, particularly when estimating the false discovery proportion becomes too variable to be reliable in sparse signal settings.

In reporting the results, unless otherwise noted, “GTS” refers to our two-step procedure using a row permuted design matrix as the regenerated and conditionally null design. For example, when Model-X knockoffs are the regenerated design, the method is called “GTS-MX”. “AGTS” refers to the two-step procedure that adjusts for the estimated proportion of nulls: ${\hat{\pi }}_0$. Logistic and Poisson simulations include the “Beta-2 Only” method, which skips the screening step in the our two-step procedure and simply applies the Benjamini–Hochberg method with target false discovery rate of $\alpha$ to the second estimate of $\varvec{\beta }$, taking advantage of the asymptotic independence: ${\hat{\beta }}_{2j} \perp \!\!\! \perp {\hat{\beta }}_{2k}$ $\forall j\ne k$.

### Logistic simulations

In the logistic regression simulations, the covariates are related to the mean of a Bernoulli distribution through the logit link function $\log (\mu _i/(1-\mu _i)) = \varvec{X}_{\varvec{i}}\varvec{\beta }$, and the response variables are drawn independently according to $Y_i \sim$ Bernoulli$(\mu _i)$ for $i = 1,...,n$. The nonzero $\beta _j$s and their signs are randomly chosen, and the signal strength increases with amplitude. The generalized two step is presented in several varieties, mainly using row permutations to regenerate the design matrix. The variant “GTS-Full Permutation,” where the regenerated design matrix is a fully randomized rearrangement of the original design, is also presented.

**Fig. 2:**
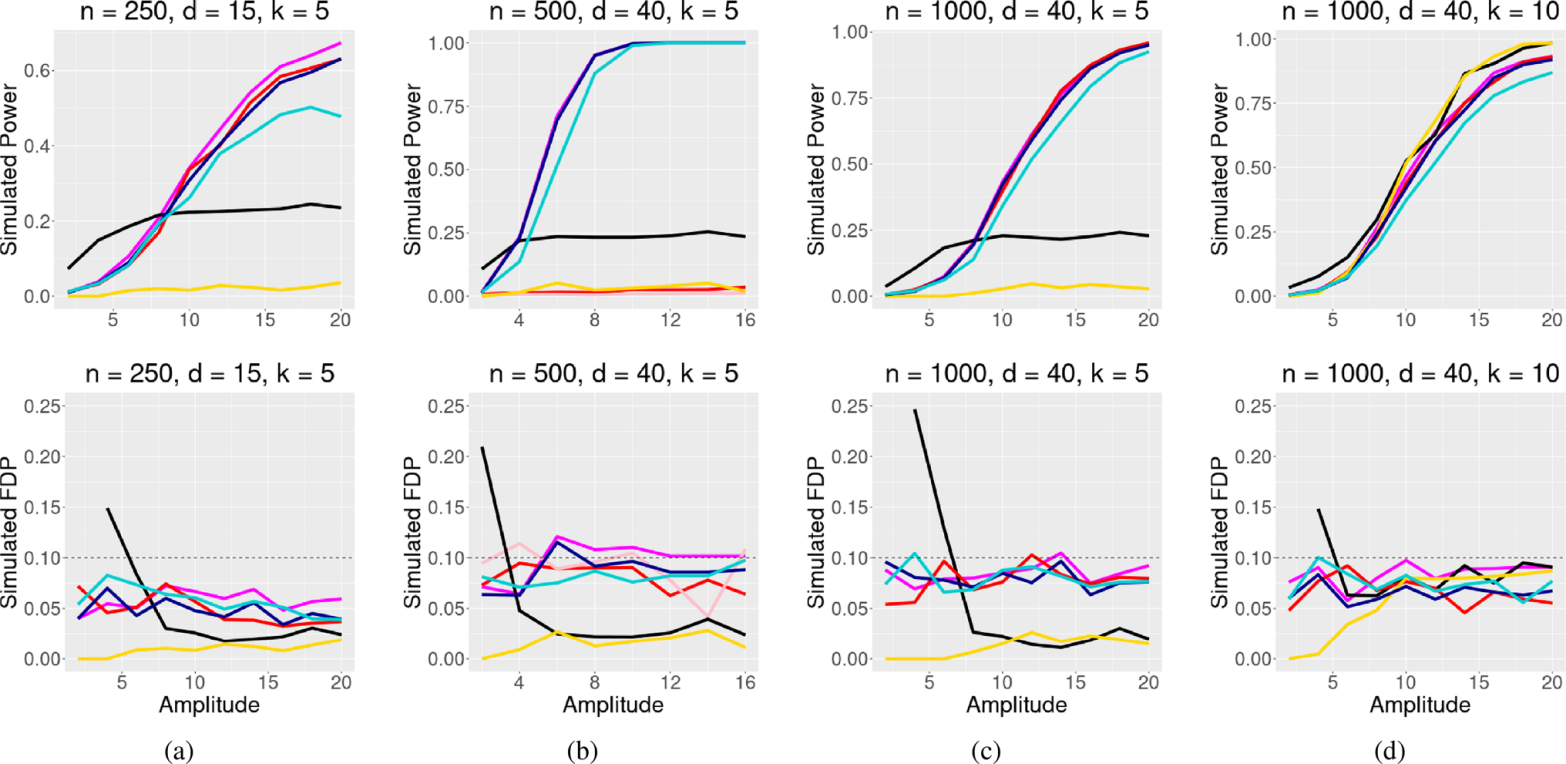
Same as Fig. 1, but $\rho$ is reduced to 0.25

In Fig. 1, the proposed method performs well in a setting with strong correlation $\rho = 0.5$. In Fig. 2, the correlation is reduced to $\rho = 0.25$, and our methods achieve higher power. The proposed two-step multiple testing method is competitive against existing methods. Also, reducing correlation improves results in settings where the proposed approach originally struggled against its competitors, as seen in Fig. 2d, compared to Fig. 1d.

### Poisson simulations

The covariates connect to the mean of a Poisson distribution through the log link function $\log (\mu _i) = X_i\varvec{\beta }$, and the outcomes $Y_i$ are drawn independently from Poisson($\mu _i$) distributions for $i = 1,\ldots ,n$. Again, the nonzero $\beta _j$s and their signs are randomly chosen, and the signal strength increases with amplitude.

**Fig. 3:**
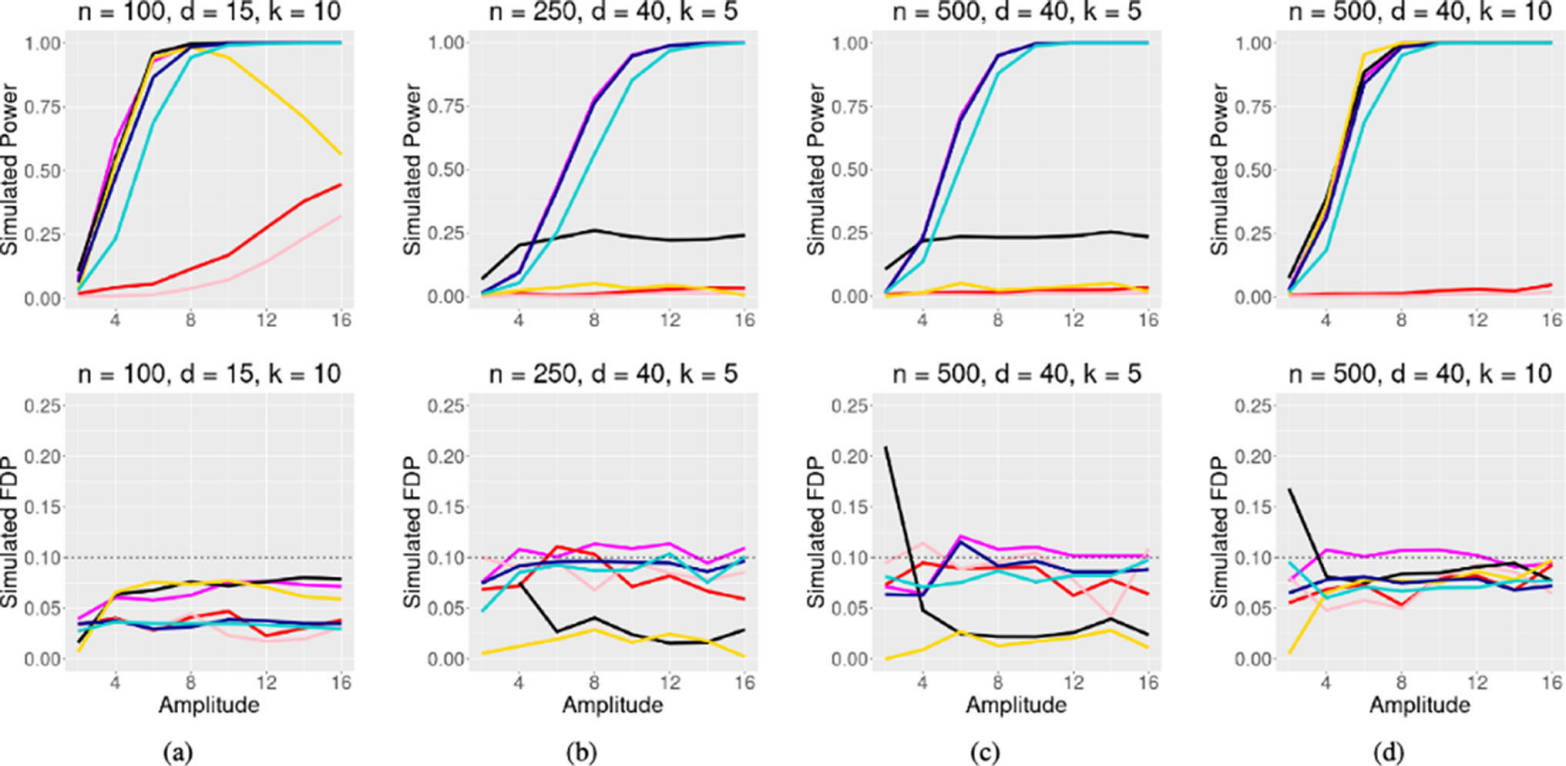
Poisson regression simulation with $\rho = 0.5$, target FDR $\alpha = 0.1$. Signal amplitude varies on the x axis, and simulated power/FDR is along the y axis. The methods compared are the GTS method, Beta-2 Only method, AGTS method,GTS-MX method, GTS-MX Beta-2 only method, **Data Splitting** (black), and the Model-X knockoff filter

**Fig. 4:**
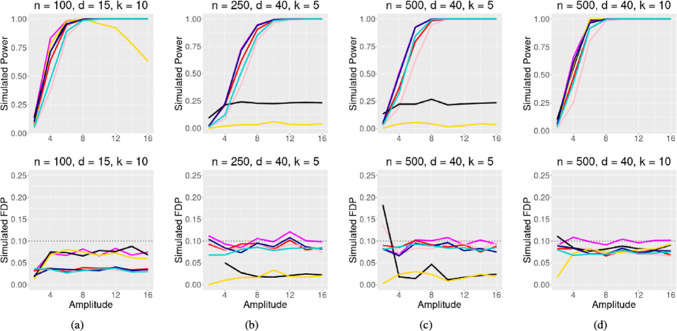
Same as Fig. 3, but with $\rho = 0.25$. Notice that GTS-MX and GTS-MX Beta-2 Only are much more competitive in this low correlation setting

In Figs. 3 and 4, four simulations are presented under strong correlation ($\rho = 0.5$), and moderate correlation ($\rho = 0.25$), respectively. Our method again performs satisfactorily in settings with sparse signal and smaller sample size, as shown in Figs. 3b, c, 4b, c. For denser signal cases (Figs. 3d, 4d), our method still performs very well.

Figures 3a and 4a display an interesting result related to the Poisson distribution. In a very dense setting where 2/3 components of $\varvec{\beta }$ are nonzeros, the Model-X knockoff filter initially performs well but then counter-intuitively loses power as the signals become stronger. This result is likely due to the mean-variance relationship of the Poisson distribution, where the variance increases linearly with its mean. As the amplitude of the coefficients increases, even with randomly assigned signs, the likelihood of observing a higher mean of *Y* and therefore a larger variance of *Y* also increases. This leads to a noisier outcome, causing the Model-X approach to be less stable.

## Real data example

The dataset employed for this analysis, originally introduced in Yeh and hui Lien (2009), is accessible through the University of California Irvine Machine Learning Repository (Yeh 2016). This dataset encompasses detailed records from clients of a major financial institution in Taiwan, focusing on whether these clients defaulted on their credit card payments in October 2005. Our empirical investigation centers on the identification of key predictors associated with credit card default risk. The predictors include a variety of demographic attributes such as gender, marital status, and education level, alongside several financial indicators. Specifically, for each month from April to September 2005, the dataset provides data on the clients’ bill amounts, payment amounts, and instances of payment delinquency. The dataset also includes the credit limit for each client. The dataset comprises 30,000 observations across 23 covariates, with a binary outcome variable. We employ logistic regression to model the default probability and perform statistical inference.

With a target FDR level of $\alpha = 0.05$, we evaluate the effectiveness of our method using two distinct types of regenerated model matrices: (i) a row-permuted model matrix, and (ii) a Model-X knockoff matrix. Additionally, we present results for a generalized version of the proposed method, as detailed in Algorithm 4 in Sect. S.2 of the supplementary material, using a row-permuted model matrix. This variant, referred to as “GTS-RP$\lambda$,” imposes generally different cutoffs on the *p*-value vectors $\varvec{P}^{({\textbf{1}})}$ and $\tilde{\varvec{P}} = (\tilde{P}_1, \ldots , \tilde{P}_d)$, instead of the common $\sqrt{\alpha }$ threshold used in Algorithm 2. The validity of this generalization is proved in Sect. S.2 of the supplementary material. The setup considered for “GTS-RP$\lambda$” presents a case for $\lambda$ near the lower boundary of the range $(\alpha ,1]$. In the screening step based on $\varvec{P}^{({\textbf{1}})}$, the $\sqrt{\alpha }$ threshold in Algorithm 2 is replaced by $1.001\alpha$ (approximately $\alpha + \epsilon ,$ where $\epsilon > 0$ is small). Rejections are then made based on $\tilde{\varvec{P}}$, where the $\sqrt{\alpha }$ threshold is replaced by $1.001^{-1}$. Our additional simulation study, reported in Sect. S.4 of the supplementary material, provides evidence of potential power improvement with this choice. The performance of our method is compared against both the Model-X knockoff filter and the Data Splitting procedure.

**Table 1:** Check marks indicate selection by the method

$\alpha = 0.05$	GTS-RP	GTS-RP$\lambda$	GTS-MX	MX	DS
Time Sept					
Bill AMT Sept					
Pay AMT Sept					
Pay AMT Aug					
Credit Limit					

The “GTS-RP$\lambda$” setup yields an average of five discoveries when using the row-permuted matrix and the generalized cutoffs. When equal cutoffs are used, the “GTS-RP” setting with the row-permuted matrix results in an average of two discoveries. In contrast, when using the Model-X matrix, the two-step procedure makes no discoveries, likely due to correlations between covariates, such as payment delinquency and bill amounts. Indeed, simulations (Figs. 3 vs. 4) show that the two-step procedure using the Model-X matrix as the re-generated design is underpowered in settings with more correlated covariates. The Data Splitting procedure selects September payment delinquency in all 250 replications but does not consistently select any other features. The Model-X knockoff filter fails to make any discoveries at the $\alpha = 0.05$ level. Table 1 showcases the variables selected by the competing methods, alongside the most frequently chosen variables. Overall, our findings demonstrate the promising performance and potential for improved power of our *p*-value-based multiple testing approach.

## Conclusion

This study introduces a novel *p*-value-based multiple testing approach tailored for generalized linear models. The method addresses the challenge of controlling the FDR in the presence of arbitrarily dependent test statistics. Theoretical analysis validates the approach, and empirical evaluations demonstrate promising performance across diverse simulation settings. The study contributes to statistical methodology by offering a flexible and computationally efficient approach for FDR control in generalized linear models with dependent test statistics.

In our current analysis, we have explored the proposed method in a conventional setting, allowing the sample size *n* to approach infinity. In practice, caution is advised when the accuracy of the distributional approximation becomes an issue, particularly when the sample size *n* is small and/or the number of covariates is moderate to large. There is also significant interest in developing new methods for controlling the FDR in high-dimensional generalized linear models, which presents substantial challenges for both methodological development and theoretical analysis. We plan to address these challenges in future work.

## Supplementary Information

Below is the link to the electronic supplementary material.

Supplementary file 1 (pdf 4329 KB)
