# Supplementary material for: A new p-value based multiple testing procedure for generalized linear models
Source: Stat Comput. 2025 Mar 16;35(3):69. doi: 10.1007/s11222-025-10600-2 (PMC11911269; doi:10.1007/s11222-025-10600-2)
Supplement: Supplementary file 1 — (pdf 4329 KB) [file 11222_2025_10600_MOESM1_ESM.pdf]

Supplementary Material

S.1 Technical detail for the construction in Section 3.2

We begin by solving for V that satisfies (4), which would make the bottom right block of the covariance matrix diagonal, and therefore ensure asymptotic independence within the second set of estimates $\hat{\boldsymbol{\beta}}_2$. Here, (4) is reprinted below:

$$J + VK^T + KV^T + VNV^T = D, \tag{4}$$

which is a nontrivial linear quadratic equation.

Let $H = D - J$, $F = -K^T$, $G = N$, (4) is then rewritten in a form of a Continuous Algebraic Riccati Equation (CARE) as (5), reprinted below

$$F^T V + VF - VGV + H = 0. \tag{5}$$

Linear quadratic equations are more often studied in control systems, where the goal is to adjust covariates to guide the behavior of a dynamic variable of interest, under some pre-specified cost function. There are some schemes available for solving CARE. In this study, we exploit the "Schur Method" of Laub (1979) in finding the stabilizing solutions of (5), as detailed below.

We begin by constructing a Hamiltonian $\Phi = \begin{bmatrix} F & -G \\ -H & -F^T \end{bmatrix} \in \mathbb{R}^{2d \times 2d}$. There exists an orthogonal transformation $U = \begin{bmatrix} U_{11} & U_{12} \\ U_{21} & U_{22} \end{bmatrix}$ such that $U^T \Phi U = S$ where S is a quasi upper triangular matrix (Murnaghan and Wintner, 1931).

$$U^T \Phi U = S = \begin{bmatrix} S_{11} & S_{12} \\ 0 & S_{22} \end{bmatrix} \tag{6}$$

Let $\lambda_k = \alpha_k + \beta_k i$, $k = 1, \ldots, 2d$, be the eigenvalues of Φ. If λ_k is real then the diagonal block is simply a 1×1 matrix $[\alpha_k]$. If the eigenvalue is complex, ($\beta_k \neq 0$), then the block on the diagonal is $\begin{bmatrix} \alpha_k & \beta_k \\ -\beta_k & \alpha_k \end{bmatrix}$, where the α_k are on the diagonal of S (Margalit and Rabinoff, 2020). This transformation is related to Schur decomposition, and is called the "Real Schur Form" (Arnold and Laub, 1984), because it allows us to have have a real output $S \in \mathbb{R}^{2d \times 2d}$. The eigenvalues of a system linearized around a fixed point determine the stability of that fixed point (Woolf, 2020). When the eigenvalues have negative real parts, the solution is stable. We can arrange S such that $S_{11} \in \mathbb{R}^{d \times d}$ corresponds to the eigenvalues of Φ that have negative real parts, and S_{22} contains the eigenvalues with positive real part. From (6), it is clear $\Phi U = US$. Then, focusing on the stabilizing solution which involves the leftmost blocks of S, particularly S_{11}, we get two equations:

$$\begin{aligned} FU_{11} - GU_{21} &= U_{11}S_{11} \\ -HU_{11} - F^T U_{21} &= U_{21}S_{11}. \end{aligned}$$

Which, after right multiplying by U_{11}^{-1}, give

$$\begin{aligned} F - GU_{21}U_{11}^{-1} &= U_{11}S_{11}U_{11}^{-1} \\ -H - F^T U_{21}U_{11}^{-1} &= U_{21}S_{11}U_{11}^{-1}. \end{aligned}$$

Then, left multiplying the upper equation by $U_{21}U_{11}^{-1}$, we get

$$\begin{aligned} U_{21}U_{11}^{-1}F - U_{21}U_{11}^{-1}GU_{21}U_{11}^{-1} &= U_{21}S_{11}U_{11}^{-1} \\ -H - F^T U_{21}U_{11}^{-1} &= U_{21}S_{11}U_{11}^{-1}. \end{aligned}$$

Therefore, we find an equality between the upper and lower equations, which can be rearranged to the original problem, where $U_{21}U_{11}^{-1}$ perfectly satisfies the role of our V in (5).

$$\begin{aligned} & U_{21}U_{11}^{-1}F - U_{21}U_{11}^{-1}GU_{21}U_{11}^{-1} \\ & \quad = -H - F^T U_{21}U_{11}^{-1} \\ \implies & F^T U_{21}U_{11}^{-1} + U_{21}U_{11}^{-1}F \\ & \quad - U_{21}U_{11}^{-1}GU_{21}U_{11}^{-1} + H = 0 \\ \implies & V = U_{21}U_{11}^{-1} \end{aligned}$$

This method has been found to consistently and appropriately solve (5) for matrices of moderate size $d \leq 100$. Publicly available programs, such as the "control" package in R by Ubah (2017) can be used for implementation. As a result, we can find V that diagonalizes the bottom right block of the covariance matrix that comes out of the linear transformation. Then, using the relationship in (3), we find P, and ultimately find the linear transformation $Z = \begin{bmatrix} I_d & P \\ I_d & V \end{bmatrix}$ such that $Z\hat{\boldsymbol{\theta}}$ gives the unbiased paired estimates for $\boldsymbol{\beta}$ that also satisfy independence constraints.

We note that the Schur method is not the only way to solve our desired Riccati equation. Guo and

Lancaster (1998) presents an alternative based off of Newton's method, and Byers (1987) introduces an approach based on the matrix sign function.

S.2 Proof of Theorem 1

We consider the conventional setting where $n > 2d$, and $n \to \infty$ while keeping d fixed. Consider a generalized linear model where the outcome $\boldsymbol{Y} = (Y_1, ..., Y_n)$ is regressed onto the augmented design matrix $(X, \tilde{X}) \in \mathbb{R}^{n \times 2d}$ through the model

$$g(\mu_i) = \eta_i = \boldsymbol{X_i}^{\mathrm{T}}\boldsymbol{\theta_1} + \boldsymbol{\tilde{X}_i}^{\mathrm{T}}\boldsymbol{\theta_2}$$

As $n \to \infty$, the maximum likelihood estimator $\hat{\boldsymbol{\theta}} = (\hat{\boldsymbol{\theta}}_{\boldsymbol{1}}^T, \hat{\boldsymbol{\theta}}_{\boldsymbol{2}}^T)^T$ approaches a normal distribution $\hat{\boldsymbol{\theta}} \sim N_{2d}((\boldsymbol{\beta}_{\boldsymbol{0}}^T, \boldsymbol{0}_{\boldsymbol{d}}^T)^T, \Omega)$, where $\boldsymbol{\beta} \in \mathbb{R}^d$ gives the true coefficients for X, and $\boldsymbol{0_d} \in \mathbb{R}^d$ is the vector of true null coefficients for the generated design matrix $\tilde{X}$, which is conditionally independent of Y given the original design X. The true covariance of the coefficients is given by $\Omega = \{(X, \tilde{X})^{\mathrm{T}} W (X, \tilde{X})\}^{-1}$, and is estimated by $\hat{\Omega}$.

Using the approach mentioned in this paper, we find the data adaptive transformation Z such that $Z\hat{\boldsymbol{\theta}} = (\hat{\boldsymbol{\beta}}_{\boldsymbol{1}}, \hat{\boldsymbol{\beta}}_{\boldsymbol{2}})^T$. As $n \to \infty$, this paired estimate is an asymptotically Gaussian random vector $Z\boldsymbol{\theta} \sim N((\boldsymbol{\beta_0}, \boldsymbol{\beta_0})^T, Z\Omega Z^T)$, where $Z\Omega Z^T$ is block diagonal, and diagonal in the lower right block. In low dimensional settings with $(2d)^{3/2} \leq n$ this approximation is generally considered appropriate (Portnoy, 1988). Therefore, in the limit, $\hat{\boldsymbol{\beta}}_1$ is independent of $\hat{\boldsymbol{\beta}}_2$, and $\hat{\beta}_{2j} \perp\!\!\!\perp \hat{\beta}_{2k} \forall j \neq k$.

Under these asymptotic constraints, $T_j^{(1)}(n)$ and $T_j^{(2)}(n)$ (with the n notation denoting the sample size) converge in distribution to independent standard Gaussians as $n \to \infty$: $T_j^{(1)}(n) \xrightarrow{d} N(0,1) \perp\!\!\!\perp T_j^{(2)}(n) \xrightarrow{d} N(0,1)$. Therefore, Algorithm 1 outputs two sets of p-values: $\boldsymbol{P^{(1)}} = \{P_j^{(1)} : j = 1, ..., d\}$ and $\boldsymbol{P^{(2)}} = \{P_j^{(2)} : j = 1, ..., d\}$, where $\boldsymbol{P^{(1)}} \perp\!\!\!\perp \boldsymbol{P^{(2)}}$, and $P_j^2 \perp\!\!\!\perp P_k^{(2)}\ \forall j \neq k$. These p-values are then used in Algorithm 2 or 3.

We present a more general form of Algorithm 2 as Algorithm 4, and then the proof of Theorem 1 under this more general setting. The general version of Algorithm 2, and the associated proof, allow the Bonferroni screening step to have cutoff λ. In the main body of the paper we set $\lambda = \sqrt{\alpha}$, but more generally λ can take any fixed value satisfying $\lambda \in (\alpha, 1]$. See Section S.4 for discussion and simulations regarding λ. When developing the proof below, the authors found Wang (2022) to be a useful reference.

It is important to note that, given $\boldsymbol{P^{(1)}}$, the $\tilde{\boldsymbol{P}} = \{\tilde{P}_j : j = 1, ..., d\}$ are conditionally independent. The $\tilde{P}_j$ either take a constant value of 1, which is trivially independent, or take the value of the corresponding $P_j^{(2)}$. The $P_j^{(2)}$ are independent thanks to the constructed covariance matrix being diagonal in the lower right block.

Proof. Let $\alpha_r = r\alpha/(\lambda d)$. The FDR of Algorithm 4, can be written as follows, where $I_0 \subseteq \{1, ..., d\}$ is the set of null variables in the model,

$$\text{FDR} = \sum_{j \in I_0} E[\mathbb{1}\{\tilde{P}_j \leq \alpha_{\tilde{R}}\}/(\tilde{R} \vee 1)]$$

This formulation can be rearranged to

$$\text{FDR} = \sum_{j \in I_0} \sum_{r=1}^{d} (1/r) E[\mathbb{1}\{\tilde{P}_j \leq \alpha_r\} \mathbb{1}\{\tilde{R} = r\}]$$

Let $\tilde{R}_{+j}$ be the number of rejections if $\tilde{P}_j = 0$ and the BH method was applied to $\tilde{\boldsymbol{P}}$. Incorporating $\tilde{R}_{+j}$, the FDR is now

$$\text{FDR} = \sum_{j \in I_0} \sum_{r=1}^{d} (1/r) E[\mathbb{1}\{\tilde{P}_j \leq \alpha_r\} \mathbb{1}\{\tilde{R}_{+j} = r\}]$$

Given $\boldsymbol{P^{(1)}}$, the $\tilde{\boldsymbol{P}}$ are independent. Each $\tilde{P}_j$ will either take a constant value of 1, which is trivially independent, or take the value of $P_j^{(2)}$, and the components of $\boldsymbol{P^{(2)}}$ are independent for all $j \neq k$. The FDR can be rewritten using the law of total expectation.

$$\text{FDR} = \sum_{j \in I_0} \sum_{r=1}^{d} (1/r) E_{\boldsymbol{P^{(1)}}} \Big\{ E[\mathbb{1}\{\tilde{P}_j \leq \alpha_r\} \mathbb{1}\{\tilde{R}_{+j} = r\} | \boldsymbol{P^{(1)}}] \Big\}$$

Because $\tilde{P}_j$ is conditionally independent of the other p-values ($\tilde{P}_{-j}$), and $\tilde{R}_{+j}$ is a function of $\tilde{P}_{-j}$, it follows that, given $\boldsymbol{P^{(1)}}$, $\tilde{P}_j \perp\!\!\!\perp \tilde{R}_{+j}$. By independence, the conditional expectation of the product within the sum is equivalent to the product of the conditional

Algorithm 4. General form of Unadjusted GTS

Input: A target FDR $\alpha \in (0,1)$, The paired p-values from Algorithm 1, Cutoff $\lambda \in (\alpha, 1]$

1. Complete a Bonferroni screening step using the first group of p-values:

$$\tilde{P}_j = \begin{cases} 1 & \text{if } P_j^{(1)} > \lambda\,, \\ P_j^{(2)} & \text{if } P_j^{(1)} \leq \lambda\,. \end{cases}$$

2. Apply the Benjamini-Hochberg method to $\tilde{P}_j$: Letting $\tilde{P}_{(1)} \leq ... \leq \tilde{P}_{(j)} \leq ... \leq \tilde{P}_{(d)}$ represent the ordered $\tilde{P}$, find

$$\tilde{R} = \max\{j : \tilde{P}_{(j)} \leq j\alpha/(\lambda d)\} \vee 0$$

3. Reject all $H_{0(j)}$ for $j \leq \tilde{R}$

expectations.

$$\text{FDR} = E_{\boldsymbol{P^{(1)}}}\{\sum_{j \in I_0}\sum_{r=1}^{d}(1/r)E[\mathbb{1}\{\tilde{P}_j \leq \alpha_r\}|\boldsymbol{P^{(1)}}]\times E[\mathbb{1}\{\tilde{R}_{+j} = r\}|\boldsymbol{P^{(1)}}]\}$$

Recall that $\tilde{P}_j = 1$ if $P_j^{(1)} > \lambda$ and $\tilde{P}_j = P_j^{(2)}$ if $P_j^{(1)} \leq \lambda$. Therefore, $\mathbb{1}\{\tilde{P}_j \leq \alpha_r\} = \mathbb{1}\{P_j^{(1)} \leq \lambda\}\mathbb{1}\{P_j^{(2)} \leq \alpha_r\}$. Furthermore, we transform the conditional expectation of the second indicator function to a conditional probability.

$$\text{FDR} = E_{\boldsymbol{P^{(1)}}}\{\sum_{j \in I_0}\sum_{r=1}^{d}(1/r)E[\mathbb{1}\{P_j^{(1)} \leq \lambda\}\times \mathbb{1}\{P_j^{(2)} \leq \alpha_r\}|\boldsymbol{P^{(1)}}]\mathbb{P}(\tilde{R}_{+j} = r|\boldsymbol{P^{(1)}})\}$$

Because the inner expectation is conditioned on $\boldsymbol{P^{(1)}}$, we can pull $\mathbb{1}\{P_j^{(1)} \leq \lambda\}$ out of the inner expectation.

$$\text{FDR} = E_{\boldsymbol{P^{(1)}}}\{\sum_{j \in I_0}\sum_{r=1}^{d}(1/r)\mathbb{1}\{P_j^{(1)} \leq \lambda\}\times E[\mathbb{1}\{P_j^{(2)} \leq \alpha_r\}|\boldsymbol{P^{(1)}}]\mathbb{P}(\tilde{R}_{+j} = r|\boldsymbol{P^{(1)}})\}$$

Because $\boldsymbol{P^{(2)}} \perp\!\!\!\perp \boldsymbol{P^{(1)}}$, we can simplify $E[\mathbb{1}\{P_j^{(2)} \leq \alpha_r\}|\boldsymbol{P^{(1)}}]$ to $\mathbb{P}(P_j^{(2)} \leq \alpha_r)$, giving

$$\text{FDR} = E_{\boldsymbol{P^{(1)}}}\{\sum_{j \in I_0}\sum_{r=1}^{d}(1/r)\mathbb{1}(P_j^{(1)} \leq \lambda)\times \mathbb{P}(P_j^{(2)} \leq \alpha_r)\mathbb{P}(\tilde{R}_{+j} = r|\boldsymbol{P^{(1)}})\}$$

Under the null, the p-values are distributed as $U(0,1)$, giving

$$\begin{aligned}\text{FDR} &= E_{\boldsymbol{P^{(1)}}}\{\sum_{j \in I_0}\sum_{r=1}^{d}(1/r)\mathbb{1}(P_j^{(1)} \leq \lambda)(\alpha_r)\mathbb{P}(\tilde{R}_{+j} = r|\boldsymbol{P^{(1)}})\} \\ &= E_{\boldsymbol{P^{(1)}}}\{\sum_{j \in I_0}\sum_{r=1}^{d}(1/r)\mathbb{1}(P_j^{(1)} \leq \lambda)\frac{r\alpha}{d\lambda}\mathbb{P}(\tilde{R}_{+j} = r|\boldsymbol{P^{(1)}})\} \\ &= E_{\boldsymbol{P^{(1)}}}\{\sum_{j \in I_0}\sum_{r=1}^{d}\mathbb{1}(P_j^{(1)} \leq \lambda)\frac{\alpha}{d\lambda}\mathbb{P}(\tilde{R}_{+j} = r|\boldsymbol{P^{(1)}})\}\end{aligned}$$

We move the sum over the variables to include only the terms with r.

$$\begin{aligned}&= E_{\boldsymbol{P^{(1)}}}\{\sum_{j \in I_0}\mathbb{1}(P_j^{(1)} \leq \lambda)\frac{\alpha}{d\lambda}\sum_{r=1}^{d}\mathbb{P}(\tilde{R}_{+j} = r|\boldsymbol{P^{(1)}})\} \\ &= E_{\boldsymbol{P^{(1)}}}\{\sum_{j \in I_0}\mathbb{1}(P_j^{(1)} \leq \lambda)\frac{\alpha}{d\lambda}\}\end{aligned}$$

Finally, simple manipulations give us asymptotic control of the false discovery rate.

$$\begin{aligned}&= \sum_{j \in I_0}\mathbb{P}(P_j^{(1)} \leq \lambda)(\alpha/(\lambda d)) = \sum_{j \in I_0}\lambda(\alpha/(\lambda d)) \\ &= \sum_{j \in I_0}(\alpha/d) = (\mid I_0 \mid)(\alpha/d) = \pi_0\alpha \leq \alpha\end{aligned}$$

This FDR control uses the asymptotic distribution. We still need to show $\lim_{n\to\infty} FDR(n) = FDR$.

The FDR is the expectation of the false discovery proportion (FDP), a function of the paired p-values $\boldsymbol{P^{(1)}}(n)$ and $\boldsymbol{P^{(2)}}(n)$. The p-values are related to the test statistics $\boldsymbol{T^{(1)}}(n)$ and $\boldsymbol{T^{(2)}}(n)$ through continuous functions. The test statistics converge in distribution to their asymptotic counterparts. Therefore, by the continuous mapping theorem, the p-values converge in distribution to their asymptotic counterparts: $\boldsymbol{P^{(1)}}(n) \xrightarrow{d} \boldsymbol{P^{(1)}}$ and $\boldsymbol{P^{(2)}}(n) \xrightarrow{d} \boldsymbol{P^{(2)}}$. As a function of the p-values, the FDP is bounded. Furthermore, the set of its discontinuities, of which there are finitely many, has measure zero. Therefore, by the portmanteau theorem,

$$\lim_{n\to\infty} E[FDP(\boldsymbol{P^{(1)}}(n),\boldsymbol{P^{(2)}}(n))] = E[FDP(\boldsymbol{P^{(1)}},\boldsymbol{P^{(2)}})]$$

In terms of the false discovery rate

$$\lim_{n\to\infty} FDR(\boldsymbol{P^{(1)}}(n),\boldsymbol{P^{(2)}}(n)) = FDR(\boldsymbol{P^{(1)}},\boldsymbol{P^{(2)}}) = \pi_0\alpha$$

Therefore $\lim_{n\to\infty} FDR(n) = \pi_0\alpha \leq \alpha$ □

S.3 Proof of Theorem 2

Maintaining the same model setup as the previous subsection, we now present a general form of Algorithm 3, and a proof of Theorem 2. The adjusted procedure estimates the proportion of null variables $\hat{\pi}_0(\eta, \boldsymbol{P^{(2)}})$. The estimate depends on a fixed $\eta \in (0, 1)$ and the values of $\boldsymbol{P^{(2)}}$. The estimated null proportion affects our adjusted $\boldsymbol{P^*}$, which differs from the $\tilde{\boldsymbol{P}}$ in unadjusted approach.

We present a more general form of Algorithm 3 as Algorithm 5, and the proof of Theorem 2 under this more general setting.

Proof. Let $\alpha_r = r\alpha/(\lambda d)$. The FDR of Algorithm 5, can be written as follows, where $I_0 \subseteq \{1, ..., d\}$ is the set of null variables in the model,

$$\begin{aligned}\text{FDR} &= \sum_{j\in I_0} E[\mathbb{1}\{P_j^* \leq \alpha_{R^*}\}/(R^* \vee 1)] \\ &= \sum_{j\in I_0} E[\mathbb{1}\{P_j^* \leq \alpha_{R^*_{+j}}\}/(R^*_{+j})]\end{aligned}$$

We begin by recognizing that $P_j^* \geq \hat{\pi}_0(\eta, \boldsymbol{P^{(2)}})\tilde{P}_j$. Construct $\hat{\pi}_{0,+j}$, the estimator of the null proportion if

---

Algorithm 5. General form of Adjusted GTS

Input: A target FDR $\alpha \in (0, 1)$, and the paired p-values from Algorithm 1, Cutoff $\lambda \in (\alpha, 1]$

1 Estimate the proportion of variables that are null using the method from Storey et al (2004) and a set $\eta \in (0, 1)$ (typically $\eta = 0.5$)

$$\hat{\pi}_0(\eta, P^{(2)}) = \frac{d - \#\{P_j^{(2)} < \eta\} + 1}{(1-\eta)d}$$

2 Complete a Bonferroni screening step using the first group of p-values:

$$P_j^* = \begin{cases} 1 & \text{if } P_j^{(1)} > \lambda\,, \\ \hat{\pi}_0(\eta, \boldsymbol{P^{(2)}})P_j^{(2)} & \text{if } P_j^{(1)} \leq \lambda \end{cases}$$

3 Apply the Benjamini-Hochberg method to P_j^*: Letting $P^*_{(1)} \leq ... \leq P^*_{(j)} \leq ... \leq P^*_{(d)}$ represent the ordered P^*, find

$$R^* = \max\{j : P^*_{(j)} \leq j\alpha/(\lambda d)\} \vee 0$$

4 Reject all $H_{0(j)}$ for $j \leq R^*$.

---

we set $P_j^{(2)} = 0$. Because $\hat{\pi}_0 \geq \hat{\pi}_{0,+j}$, we obtain the following order of inequalities

$$P_j^* \geq \hat{\pi}_0(\eta, \boldsymbol{P^{(2)}})\tilde{P}_j \geq \hat{\pi}_{0,+j}\tilde{P}_j$$

These inequalities allow us to put an upper bound on the FDR

$$\text{FDR} \leq \sum_{j\in I_0} E[\mathbb{1}\{\hat{\pi}_{0,+j}\tilde{P}_j \leq \alpha_{R^*_{+j}}\}/(R^*_{+j})]$$

We now rearrange the right hand side as shown in the supplementary material of Sarkar and Tang (2022) to obtain the following, where we mandate that $0/0 = 0$ and $\alpha_0 = 0$

$$\text{FDR} \leq \sum_{j\in I_0}\sum_{r=1}^{d} E\Bigg[\mathbb{P}(R^*_{+j} \geq r, P_j^{(1)} \leq \lambda)\times \left[\frac{\mathbb{1}(\hat{\pi}_{0,+j}P_j^{(2)} \leq \alpha_r)}{r} - \frac{\mathbb{1}(\hat{\pi}_{0,+j}P_j^{(2)} \leq \alpha_{r-1})}{r-1}\right] \mid P^{(1)}_{-j}\Bigg]$$

Because $\boldsymbol{P^{(1)}} \perp\!\!\!\perp \boldsymbol{P^{(2)}}$, the condition can be moved within the expectation

$$\text{FDR} \leq \sum_{j \in I_0} \sum_{r=1}^{d} E\Bigg[\mathbb{P}(R^*_{+j} \geq r, P^{(1)}_j \leq \lambda | P^{(1)}_{-j}) \times \left[\frac{\mathbb{1}(\hat{\pi}_{0,+j} P^{(2)}_j \leq \alpha_r)}{r} - \frac{\mathbb{1}(\hat{\pi}_{0,+j} P^{(2)}_j \leq \alpha_{r-1})}{r-1}\right]\Bigg]$$

and can be re-written as

$$\text{FDR} \leq \sum_{j \in I_0} \sum_{r=1}^{d} E\Bigg[\mathbb{P}(R^*_{+j} \geq r \mid P^{(1)}_{-j}) \mathbb{P}(P^{(1)}_j \leq \lambda | P^{(1)}_{-j}) \left[\frac{\mathbb{1}(\hat{\pi}_{0,+j} P^{(2)}_j \leq \alpha_r)}{r} - \frac{\mathbb{1}(\hat{\pi}_{0,+j} P^{(2)}_j \leq \alpha_{r-1})}{r-1}\right]\Bigg]$$

Now, because $\hat{\pi}_0(\eta, \boldsymbol{P^{(2)}})$ is component wise increasing in $\boldsymbol{P^{(2)}}$, the aforementioned equation can be bounded above by conditioning $\mathbb{P}(R^*_{+j} \geq r \mid P^{(1)}_{-j})$ on $\hat{\pi}_{0,+j} P^{(2)}_j$ such that $\mathbb{P}(R^*_{+j} \geq r \mid P^{(1)}_{-j}, \hat{\pi}_{0,+j} P^{(2)}_j = \alpha_{r-1})$. This conditioning gives us the following inequality

$$\text{FDR} \leq \sum_{j \in I_0} E\Bigg[\sum_{r=1}^{d} \mathbb{P}(R^*_{+j} \geq r \mid P^{(1)}_{-j}, \hat{\pi}_{0,+j} P^{(2)}_j = \alpha_{r-1}) \times \mathbb{P}(P^{(1)}_j \leq \lambda | P^{(1)}_{-j}) \times \left[\frac{\mathbb{P}(\hat{\pi}_{0,+j} P^{(2)}_j \leq \alpha_r)}{r} - \frac{\mathbb{P}(\hat{\pi}_{0,+j} P^{(2)}_j \leq \alpha_{r-1})}{r-1}\right]\Bigg]$$

Under the null hypothesis the fraction $(\mathbb{P}(\hat{\pi}_{0,+j} P^{(2)}_j \leq \alpha_r | P^{(2)}_{-j}))/r = \min\{(1/r), \alpha/(\lambda d \hat{\pi}_{0,+j})\} = \min\{(1/r), \alpha_1/\hat{\pi}_{0,+j})\}$, and is decreasing in r. Therefore, again enforcing the condition $0/0 = 0$, which is important for the differences when $r = 1$, the sum of the differences has an upper bound of $\alpha_1/\hat{\pi}_{0,+j}$.

$$\begin{aligned}
\text{FDR} &\leq \sum_{j \in I_0} E\Bigg[\sum_{r=1}^{d} \mathbb{P}(R^*_{+j} \geq r \mid P^{(1)}_{-j}, \hat{\pi}_{0,+j} P^{(2)}_j = \alpha_{r-1}) \\
&\quad \times \mathbb{P}(P^{(1)}_j \leq \lambda | P^{(1)}_{-j}) \\
&\quad \times \left[\frac{\mathbb{P}(\hat{\pi}_{0,+j} P^{(2)}_j \leq \alpha_r)}{r} - \frac{\mathbb{P}(\hat{\pi}_{0,+j} P^{(2)}_j \leq \alpha_{r-1})}{r-1}\right]\Bigg] \\
&\leq \sum_{j \in I_0} E\Bigg[\mathbb{P}(P^{(1)}_j \leq \lambda | P^{(1)}_{-j})(\alpha_1/\hat{\pi}_{0,+j}) \\
&\quad \times \sum_{r=1}^{d} \mathbb{P}(R^*_{+j} \geq r \mid P^{(1)}_{-j}, \hat{\pi}_{0,+j} P^{(2)}_j = \alpha_{r-1})\Bigg] \\
&\leq \sum_{j \in I_0} E\Big[\mathbb{P}(P^{(1)}_j \leq \lambda | P^{(1)}_{-j})(\alpha_1/\hat{\pi}_{0,+j})\Big] \\
&= \lambda \alpha_1 \sum_{j \in I_0} E\Big[1/\hat{\pi}_{0,+j}\Big] \\
&\leq \lambda \alpha_1 d = \alpha
\end{aligned}$$

We proved this inequality utilizing the asymptotic distribution. We can move to the statement $\lim_{n \to \infty} FDR(n) = FDR \leq \alpha$ by using the portmanteau theorem and the argument presented at the end of the proof of Theorem 1 in Section S.2, with the addendum that the estimate of the null proportion is also a function of the p-values whose discontinuities has measure zero. □

S.4 Exploring the role of λ

In the generalized version of our proposed method, we add another parameter $\lambda \in (\alpha, 1]$, to the two step procedure. This λ controls the severity of the cutoffs for the p-values of each stage. Higher values of λ lead a looser screening step, but more rigorous cutoffs for the second step based on $\tilde{\boldsymbol{P}}$. Lower values of λ create a stricter screening step, but the cutoffs for the second step are less punishing. Setting $\lambda = 1$ gives the Beta-2 Only method discussed in the paper. Setting $\lambda = \alpha + \epsilon$, where $\epsilon > 0$ is small, is an interesting scenario. Effectively, screening based on $\boldsymbol{P^{(1)}}$ is not corrected for multiple testing, and then the second step is akin to a Benjamini Hochberg test aiming for a target FDR of almost 1. Still, the two steps together control the FDR.

We provide simulations to investigate the role of λ below. These simulations are presented in Figures 5, 6, and 7. To more easily examine the role of λ we introduce a variable $w \in (0, 1]$, and define $\lambda = \alpha^{1-w}$. Because $\alpha \in (0, 1)$, λ is strictly increasing in w. Setting $w = 1$ corresponds to $\lambda = 1$, the maximum value, and gives the Beta-2 only procedure. Setting $w > 0$ close to zero gives $\lambda \approx \alpha + \epsilon$, the second procedure discussed above. We plot the estimated power and false discovery proportion of our method on the y-axis against w which increases along the x-axis. The coefficient of correlation is set to $\rho = 0.5$, and the target FDR is set to $\alpha = 0.1$. The various lines indicate

different levels of signal strength, defined though the amplitude parameter of Section 4, and the simulations are repeated 250 times.

Figure 5 gives the results for logistic regression. In logistic regression $\lambda = \sqrt{\alpha}$, as proposed in the main body of our paper, appears optimal. For the Poisson simulations in Figure 6 and the linear simulations in Figure 7, setting $w = 0.001$, giving $\lambda = \alpha^{0.999} \approx 0.1002 \approx \alpha+\epsilon$ is typically the most powerful. Furthermore, as we claim in our generalized algorithm and accompanying proofs, the false discovery rate is controlled for all $w \in (0, 1]$ and corresponding $\lambda \in (\alpha, 1]$, with $\lambda = 1$ giving the "Beta-2 Only" procedure.

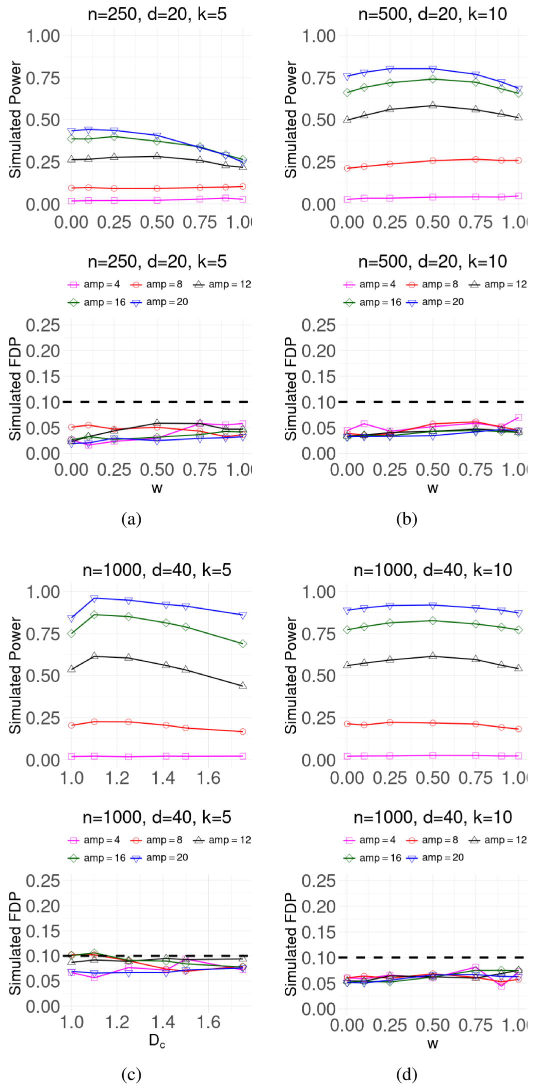

Fig. 5: Logistic regression simulations where $\lambda = \alpha^{1-w}$ is varied along the x-axis. Define $\lambda = \alpha^{1-w}$. Because $\alpha \in (0, 1)$, λ is strictly increasing in w. The highest power is achieved around $w = 0.5$, giving $\lambda = \sqrt{\alpha}$, which is used in the main paper. The false discovery rate is always controlled.

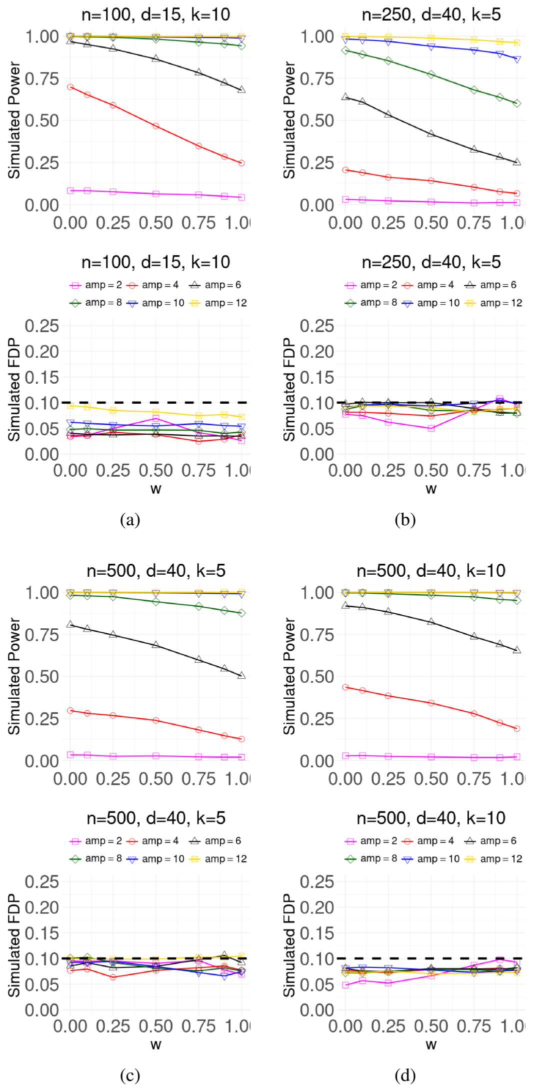

Fig. 6: Poisson regression simulations where $\lambda = \alpha^{1-w}$ is varied along the x-axis. Power appears to be decreasing with λ. The false discovery rate is always controlled.

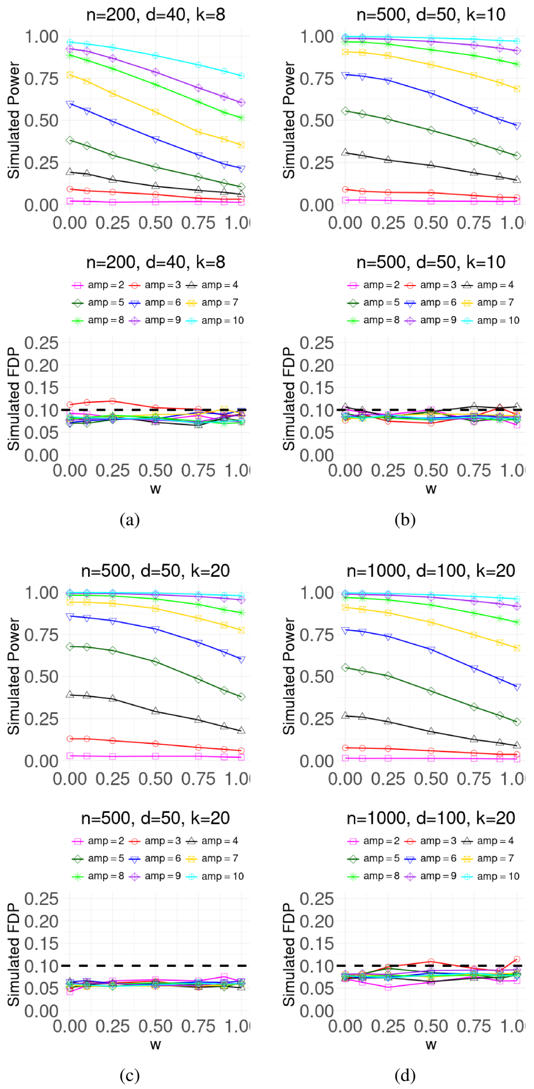

Fig. 7: Linear regression simulations where $\lambda = \alpha^{1-w}$ is varied along the x-axis. Power appears to be decreasing with λ. The false discovery rate is always controlled.

S.5 Exploring the choice of D

The diagonal matrix D gives the variance of the second estimate $\hat{\boldsymbol{\beta}}_{\mathbf{2}}$, and can be specified by the user, subject to the condition that $D-J$ is positive semidefinite. This choice provides the user with great flexibility, but may also be daunting. In Section 3.2, we suggest that decision makers set the entries of D to a constant multiple $\delta_c \geq 1$ of $\lambda_{J,\max}$, the largest eigenvalue of J, giving $D = \text{diag}(\delta_c \times \lambda_{J,\max})$. We further suggest a default factor of $\delta_c = 1.1$, which we find and support through the simulation study presented in Figures 8, 9, and 10. The graphs plot the estimated power and false discovery proportion of the generalized two step on the y-axis against δ_c, the multiplicative constant, which increases along the x-axis. The coefficient of correlation is set to $\rho = 0.5$. The target FDR is $\alpha = 0.1$. The various lines indicate different levels of signal strength, defined through the amplitude parameter from Section 4, and the simulations are repeated 250 times.

From these simulations, it is clear that a lower δ_c is generally better. In linear and Poisson simulations, the power is strictly decreasing in δ_c. The multiplicative constant $\delta_c = 1.1$ performs well in all considered settings, and is therefore our suggested default. In summary, D controls the variance of the second set of coefficients, and it makes sense that lower variances correspond to increased power.

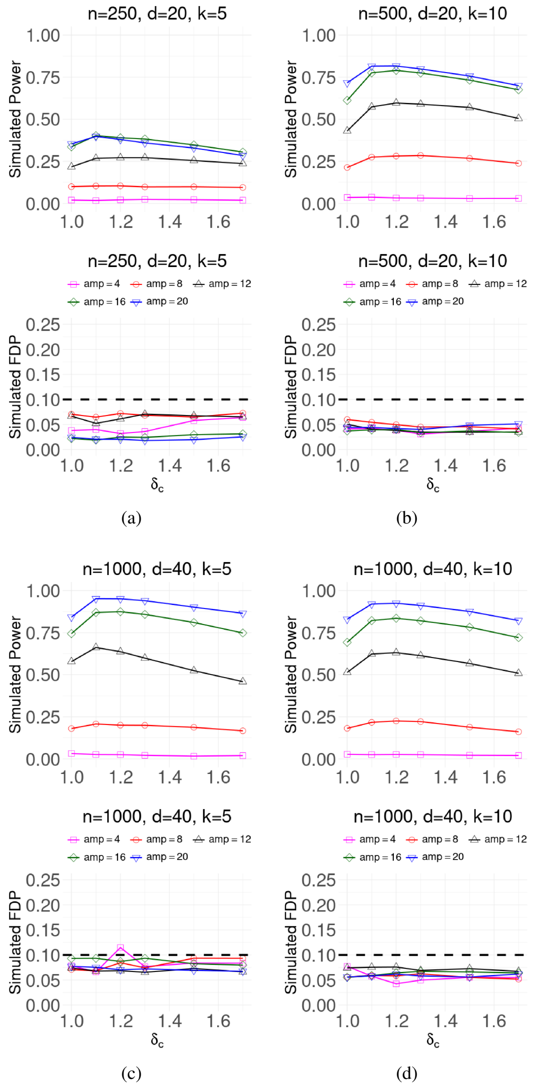

Fig. 8: Logistic regression simulations where the multiplicative constant δ_c is varied along the x-axis. The power appears highest at $\delta_c = 1.1$, and then steadily declines as δ_c increases. The false discovery rate is always controlled.

S.6 Non Existence of an Adequate Static Transformation

The BBH method (Sarkar and Tang, 2022) uses fixed-X knockoffs ($\tilde{X}_F$) and a non-adaptive linear transformation $A = \begin{bmatrix} I_d & I_d \\ I_d & -I_d \end{bmatrix}$, but for cases outside of homoscedastic linear regression, this approach no longer guarantees the useful properties about dependence between the estimates. For generalized linear models with large samples, the maximum likelihood estimator is approximately distributed as:

$$\hat{\boldsymbol{\theta}} \sim N(\boldsymbol{\theta}, ((X, \tilde{X}_F)^T \hat{W} (X, \tilde{X}_F))^{-1})$$

$\hat{W}$ is a constant $\hat{\sigma}^{-2} I_n$ in homoscedastic linear regression. Therefore, $\hat{W}$ can be pulled out in front of the rest of the covariance matrix, and the known values of $(X, \tilde{X}_F)^T (X, \tilde{X}_F)$ can be used to find the matrix A that satisfies our desired properties, independent of the outcome $\boldsymbol{Y}$. In most generalized linear models, $\hat{W}$ is not a constant diagonal and cannot be removed.

Suppose as before that we find the estimated covariance of $\hat{\boldsymbol{\theta}}$ is $((X, \tilde{X}_F)^T \hat{W} (X, \tilde{X}_F))^{-1} = \begin{bmatrix} J & K \\ K^T & N \end{bmatrix}$, where each block is d by d, and J and N are symmetric. If we transform $\hat{\boldsymbol{\theta}}$ to $A\hat{\boldsymbol{\theta}}$, then the covariance becomes:

$$A \begin{bmatrix} J & K \\ K^T & N \end{bmatrix} A^T = \begin{bmatrix} (J+K^T)+(K+N) & (J+K^T)-(K+N) \\ (J-K^T)+(K-N) & (J-K^T)-(K-N) \end{bmatrix}$$

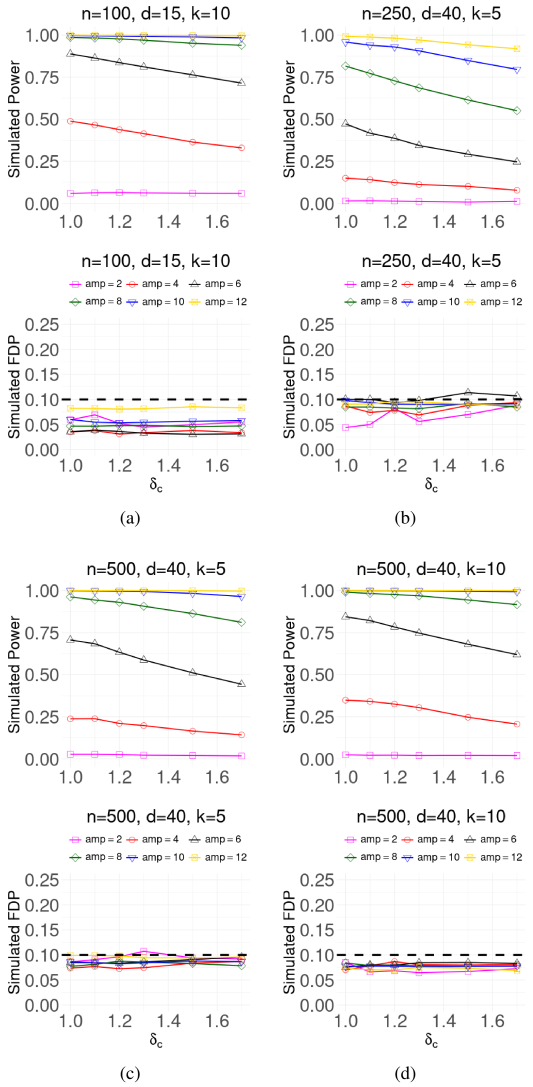

Fig. 9: Poisson regression simulations where the multiplicative constant δ_c is varied along the x-axis. The power appears to be steadily decreasing as δ_c increases. The false discovery rate is always controlled.

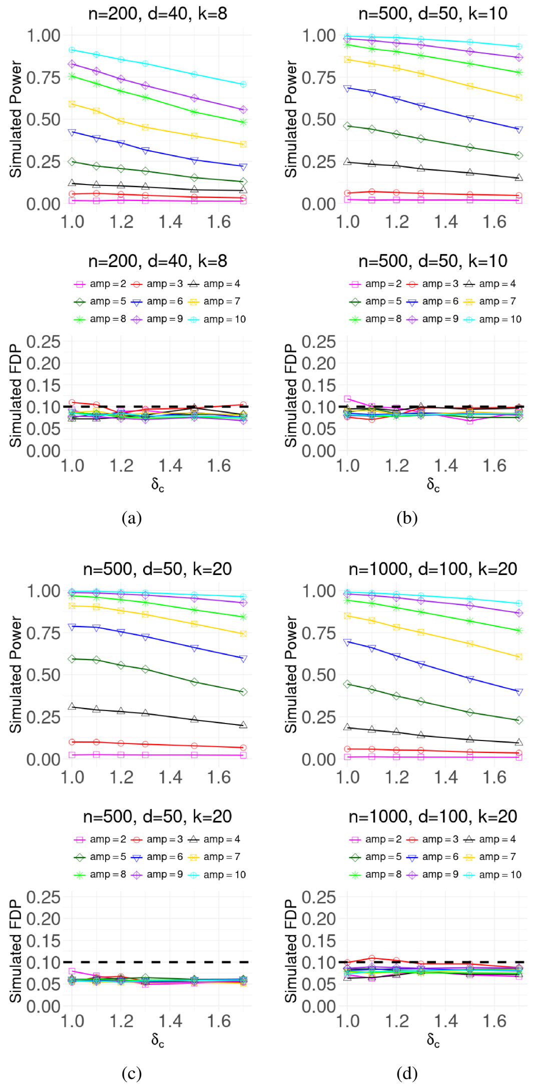

Fig. 10: Linear regression simulations where the multiplicative constant δ_c is varied along the x-axis. The power appears to be steadily decreasing as δ_c increases. The false discovery rate is always controlled.

Now, if we want $\hat{\boldsymbol{\beta}}_1 \perp\!\!\!\perp \hat{\boldsymbol{\beta}}_2$, we need $J + K^T = K + N$. For independence within $\hat{\boldsymbol{\beta}}_2$, we need $J - K^T - K + N$ to be diagonal. These conditions hold for standard linear models, but do not hold for generalized linear models. In fact, due to the variation in W, neither the original transformation A, nor any other pre-set static transformation guarantees the necessary independence conditions.

S.7 Additional Simulations with Linear Models

In this section we present three simulation studies. In Figure 11, the target FDR is set to $\alpha = 0.05$. The BBH method holds a slight edge over the proposed GTS, but both methods dominate the Fixed-X and Model-X knockoff filters. When the FDR is raised to $\alpha = 0.1$

in Figure 12, the knockoff approaches are more competitive in the dense, large sample environments. In both Figure 11 and Figure 12, the coefficient of correlation is $\rho = 0.5$. Figure 13 displays the power and FDR of various methods vs. an increasing ρ on the x-axis. The peach colored line uses Model-X knockoffs as the generated input for GTS. This method demonstrates a drastic drop off in power when the covariates become more correlated, even though the Model-X knockoff's performance remains stable. The pink line retains Model-X knockoffs as the generated design, but uses the static transformation from BBH. This modified BBH method (using Model-X knockoffs instead of Fixed-X knockoffs) is appropriate in the limit and shows more power than the GTS method, likely due to some nice properties associated with A, but still struggles with highly correlated settings. Therefore, we do not believe that the transformation (A vs. Z) is causing the issue, but instead conclude that the Model-X knockoffs can be a poor synthetic input for correlated settings. The dark blue line uses a row permutation as the regenerated $\tilde{X}$ input for GTS. With a more suitable $\tilde{X}$ chosen, we observe the GTS method retains more power in correlated settings.

In linear regression, there is also a special case of the GTS method that uses a more direct solution for Z instead of using the Schur method. Because the weight matrix in linear regression is constant $W = \hat{\sigma}^{-2} I_n$, this term can be removed from the middle of the covariance matrix

$$\Omega = \left[(X, \tilde{X})^T W (X, \tilde{X})\right]^{-1} = \hat{\sigma}^2 \left[(X, \tilde{X})^T (X, \tilde{X})\right]^{-1}$$

Therefore, in linear regression selecting a null $\tilde{X}$ such that $(X, \tilde{X})^T (X, \tilde{X})$ is known permits an analytical solution for the linear transformation Z, that does not rely on the schur method and control theory. For example, the BBH method utilizes $\tilde{X}_F$ because the combined covariance matrix is known.

$$(X, \tilde{X}_F)^T (X, \tilde{X}_F) = \begin{bmatrix} \Sigma & \Sigma - D \\ \Sigma - D & \Sigma \end{bmatrix} \in \mathbb{R}^{2d \times 2d}$$

This covariance matrix also leads nicely to a Z whose rows/columns are orthogonal, and is a constant independent of X. A special extension similarly applies for $\tilde{X}_P$, where rows in X are permuted. Under this permutation, the covariance matrix is the inverse of a known block diagonal:

$$\left[(X, \tilde{X}_P)^T (X, \tilde{X}_P)\right]^{-1} = \begin{bmatrix} \Sigma & 0 \\ 0 & \Sigma \end{bmatrix}^{-1} = \begin{bmatrix} \Sigma^{-1} & 0 \\ 0 & \Sigma^{-1} \end{bmatrix}$$

In the special case of row permutations, $Z = \begin{bmatrix} I_d & P \\ I_d & V \end{bmatrix}$, where $V = (D - \Sigma^{-1})^{1/2} \Sigma^{1/2}$ and $P = -\Sigma^{-1} [V^T]^{-1} \Sigma$, gives a valid data dependent transformation that adequately prepares for a two step procedure. This "GTS-Analytical" method is presented throughout the linear simulations. The closed form special case is more tractable but is still data dependent because Z relies on Σ. This dependency further emphasizes the unique setup and payoff in Sarkar and Tang (2022). Also, the "GTS-Analytical" approach does not outperform our default generalized two step using the Schur method, suggesting that the default method from the main body of the paper is adequately estimating Z.

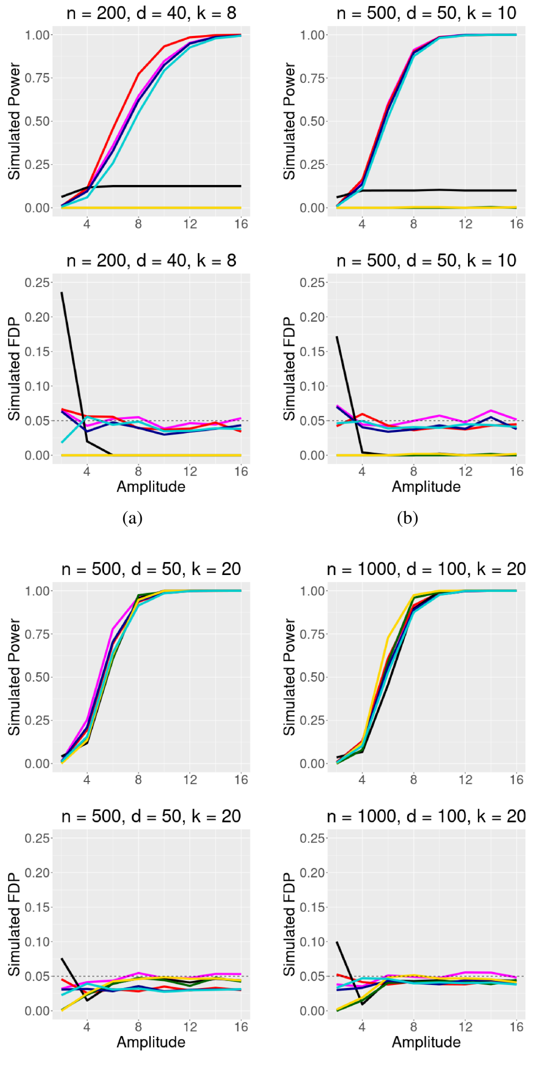

(a) (b) (c) (d)

Fig. 11: Linear regression simulation with target FDR $\alpha = 0.05$, The methods compared are the BBH method, AGTS method, GTS method, GTS-Analytical, **Data Splitting**, and the Model-X and Fixed-X knockoff filters

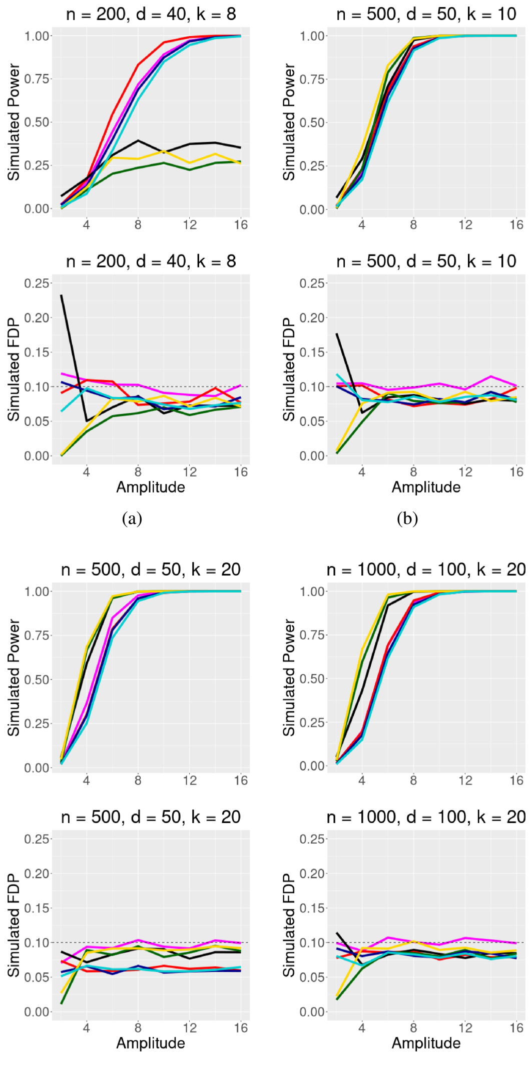

(a) (b) (c) (d)

Fig. 12: Same as Figure 11, but $\alpha = 0.1$

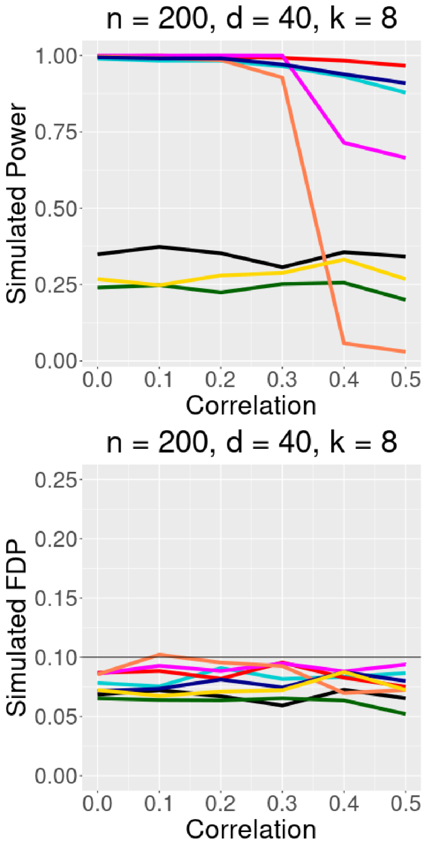

Fig. 13: Linear regression simulation with amplitude 10 and target FDR $\alpha = 0.1$. Covariate correlation ρ varies on the x axis from 0 to 0.5, and simulated power/FDR is along the y axis. The methods compared are the BBH method, BBH-MX method, GTS-MX method, GTS-Row Permutation method, GTS Analytical, Data Splitting (black), and the Model-X and Fixed-X knockoff filters.

References

Arnold W, Laub A (1984) Generalized eigenproblem algorithms and software for algebraic riccati equations. Proceedings of the IEEE 72(12):1746–1754. https://doi.org/10.1109/PROC.1984.13083

Byers R (1987) Solving the algebraic riccati equation with the matrix sign function. Linear Algebra and its Applications 85:267–279. https://doi.org/10.1016/0024-3795(87)90222-9, URL https://www.sciencedirect.com/science/article/pii/0024379587902229

Guo CH, Lancaster P (1998) Analysis and modification of newton's method for algebraic riccati equations. Mathematics of Computation 67(223):1089–1105. URL https://www.jstor.org/stable/2585172

Laub A (1979) A Schur method for solving algebraic Riccati equations. IEEE Transactions on Automatic Control 24(6):913–921. https://doi.org/10.1109/TAC.1979.1102178, URL http://ieeexplore.ieee.org/document/1102178/

Margalit D, Rabinoff J (2020) Interactive Linear Algebra, pdf version edn., chap Complex Eigenvalues

Murnaghan FD, Wintner A (1931) A canonical form for real matrices under ortho-gonal transformations. Proceedings of the National Academy of Sciences of the United States of America 17(7):417–420. URL http://www.jstor.org/stable/86181

Portnoy S (1988) Asymptotic Behavior of Likelihood Methods for Exponential Families when the Number of Parameters Tends to Infinity. The Annals of Statistics 16(1):356 – 366. https://doi.org/10.1214/aos/1176350710, URL https://doi.org/10.1214/aos/1176350710

Sarkar SK, Tang CY (2022) Adjusting the Benjamini–Hochberg method for controlling the false discovery rate in knockoff-assisted variable selection. Biometrika 109(4):1149–1155. https://doi.org/10.1093/biomet/asab066, URL https://doi.org/10.1093/biomet/asab066, https://academic.oup.com/biomet/article-pdf/109/4/1149/47415774/asab066_supplementary_data.pdf

Storey JD, Taylor JE, Siegmund D (2004) Strong Control, Conservative Point Estimation and Simultaneous Conservative Consistency of False Discovery Rates: A Unified Approach. Journal of the Royal Statistical Society Series B: Statistical Methodology 66(1):187–205. https://doi.org/10.1111/j.1467-9868.2004.00439.x, URL https://doi.org/10.1111/j.1467-9868.2004.00439.x, https://academic.oup.com/jrsssb/article-pdf/66/1/187/49727331/jrsssb_66_1_187.pdf

Ubah BC (2017) control: A Control Systems Toolbox. URL https://CRAN.R-project.org/package=control, r package version 0.2.5

Wang R (2022) Elementary proofs of several results on false discovery rate. 2201.09350

Woolf P (2020) Using Eigenvalues and Eigenvectors to Find Stability and Solve ODEs. URL https://eng.libretexts.org/@go/page/22502, publisher: University of Michigan
